# The Descriptions and Attitudes of Riders and Arena Owners to 656 Equestrian Sport Surfaces in Sweden

**DOI:** 10.3389/fvets.2021.798910

**Published:** 2021-12-23

**Authors:** Agneta Egenvall, Lars Roepstorff, Michael Peterson, Marcus Lundholm, Elin Hernlund

**Affiliations:** ^1^Department of Clinical Sciences, Faculty of Veterinary Medicine and Animal Husbandry, Swedish University of Agricultural Sciences, Uppsala, Sweden; ^2^Department of Anatomy, Physiology and Biochemistry, Faculty of Veterinary Medicine and Animal Husbandry, Swedish University of Agricultural Sciences, Uppsala, Sweden; ^3^Biosystems and Agricultural Engineering, College of Agriculture, Food and Environment, University of Kentucky, Lexington, KY, United States; ^4^Department for Riding Schools, Swedish Equestrian Federation, Ridsportens Hus, Strömsholm, Sweden

**Keywords:** horse, arena composition, sand particles, sand-fibre, woodchips, surface maintenance

## Abstract

Horses in equestrian sports are commonly trained in arenas with prepared footing. Information on the number and variants of such arenas is generally unknown. This paper provides an overview of the primary construction types of riding surfaces in Sweden including details on composition, constructions principles, usage frequency, maintenance, and cost of operation as well as to investigate rider perception of the ideal arena properties using a large population of riders. Data on 656 equestrian surfaces in Sweden obtained up to 2014 are presented, of which 373 were outdoor and 283 were indoor arenas. Dressage and show-jumping were the main disciplines conducted in the arenas. Sand-mineral arenas were most common outdoors and sand-woodchips arenas most common indoors, followed by sand-fibre arenas and even fewer synthetic arenas. Comparing the three most common arena types, dragging was most often done on sand-woodchips and sand-fibre arenas. Harrowing was less often done on sand-mineral arenas compared to sand-woodchips and sand-fibre arenas. Combining dragging, harrowing, deep harrowing, and rolling, arenas with higher usage were maintained more frequently, compared to those used less frequently. It was commonly claimed that the top-layer needs renovation every other-4th year or every 5th to 10th year. Few respondents allocated more than 10,000 SEK in yearly maintenance costs, with the exception for sand-woodchips and sand-fibre arenas followed by synthetic arenas. The shortest duration perceived between required renovations was found for sand-woodchips top-layer arenas. Ideal surface properties were evaluated by 3,158 riders. Dressage and show-jumping riders differed somewhat regarding ideal spans of functional arena properties: for impact firmness, responsiveness, and grip. The current study likely included well-utilised arenas, compared to those less well-utilised. The resources necessary to keep an arena consistent over time seemed underestimated. Knowledge of maintenance and priorities for arenas are important to users and arenas managers, be they construction companies or arena managers in order to maximise the outcome of efforts for arena improvement and optimise locomotor health for horses that use them. Further, many arenas were new and research into organic arena management is important, especially if equestrians continue to build and renew arena surfaces.

## Introduction

The training and competition surfaces used by horses have gained increasing interest from the equine industry, from scientists, and from sports governing bodies over the last decade. This is warranted since the surface has been identified as a risk factor for injuries (1–4) and affects performance in the equine athlete (5). Evidence-based recommendations for surface construction and maintenance are needed in order to minimise sport related injuries in horses. The first step in this process is to understand what types of surfaces are currently in use and how they are maintained.

The efforts to provide safe surfaces have hitherto mainly been focused on the top level of the equestrian sports (6), where fair competition is a high priority. In order to maintain a social licence for the sport, it is necessary to keep the welfare of the horse a main priority, and catastrophic injuries are devastating for the reputation of the sport. Functional properties of riding surfaces have been defined (7) to enable quantification of the surface response to the loading of a horse and how the rider perceives the surface performance. Surface testing devices have been developed to measure these properties (i.e., the surface's mechanical behaviour) in order to help guide the process of quality assurance of equine sport surfaces (6, 8, 9).

The goal of providing recommendations regarding construction and maintenance must of course extend beyond the very top equine athletes. The majority of horses are not trained on the type of surfaces seen at high level competitions. Many horses used in riding schools and for leisure riding experience sustain high volumes of repetitive work on one or a few arena surfaces. They are thus likely to have a high level of exposure to the risks associated to poor surface properties. If the scientific community wishes to provide evidence-based recommendations, which can reduce the incidence of surface related injuries, some basic facts regarding composition, maintenance, and resources must be known about the arena surfaces that a majority of horses are being exercised on. This information is currently not available.

The material composition of the surface has been shown to be a risk-factor for injuries in Thoroughbred race horses in numerous epidemiological studies from several continents (10). Field experts report a great variation of materials used in surfaces for riding sports across the world (7). Systematic information regarding composition and construction of arenas is however missing. The main prevailing construction principles incorporate a loose upper layer (most often sand), allowing motion of the hoof early in the stance phase, and an underlying firm base for support (11). Local preferences are also evident (4) and are possibly governed by climate conditions, the quality of local granular minerals (sands) and the access to suitable residual products from local industry. The residual products can be woodchips or sawdust, cloth strips, polymer fibres, rubber particles, PVC pieces and paraffin-based high-oil content waxes. These products are commonly mixed with the sand in the top-layer in different proportions (7, 12). Surfaces that include materials with a polymeric component are classified as synthetic surfaces (13). These surfaces (e.g., sand-fibre surfaces, waxed sand surfaces) have gained popularity in the equine industry over the last two decades. Concern regarding their effect on the orthopaedic soundness of riding horses have been raised (14). So far, the few studies conducted to identify risks associated to different types of arena surfaces (3, 4) have not shown results supporting this concern. The absence of work which associates injury with types of surfaces makes it difficult to understand the field. In addition, geographical differences in surfaces and changes in types of surfaces used over time will have to be considered.

Taken together, the possible importance of arena surfaces for the health and performance of the general riding horse, and the substantial economic resources we anticipated were spent on new and old arenas motivated a cross-sectional study of riding arenas in Sweden. Such a study would provide a picture of a large number of arenas and their construction details, enabling further research into equestrian surfaces not only at the top-level. The first objective was to provide an overview of the construction of riding surfaces including details regarding the composition, constructions principles, the usage frequency, maintenance and the economic implications, especially the cost over time or total cost of ownership. The second objective was to investigate rider perception of ideal arena properties in a larger population of Swedish riders.

## Materials and Methods

### Design

In April 2014 an internet questionnaire was distributed by the Swedish Equestrian Federation (SvRF) to riders and to larger riding establishments in Sweden using Netigate (15). The Swedish Equestrian Federation is a governing body for the equestrian sports in Sweden, disregarding the Icelandic horse sports that have a body of their own. There are ~900 associations covered by SvFR, of which 50% are riding schools and 50% are other associations most often arranging competitions. The questionnaire was sent to all 15,356 riders that competed at intermediate level or higher in show-jumping, dressage, or eventing and thus were registered with the SvRF as competition riders, as well as to 891 establishments with riding arenas. The latter list was derived from the SvRF riding club member register. At the establishment the questionnaire was targeted specifically to personel in charge of the riding arenas. The answers were treated unidentified. Because of lack of official data it is difficult to state exact what proportions are covered by SvRFs registries, but to the best of our perception the coverage of targeted arenas approaches 80% and of competing riders at the stated level 100%. An ethical permit was not considered necessary for this type of study according to Swedish law.

### The Questionnaire

Translated version of how questions were posed are found in Supplementary Materials 1, 2. Both multiple choice and free-text questions were included. The rider questionnaire contained two parts. In the first part riders were asked to rate how they wanted the characteristics of the riding arenas for competition purposes and while training. The characteristics were impact firmness, cushioning, responsiveness, grip, and uniformity [(6, 7), definitions see Supplementary Material 1]. The second part, which for this latter part was identical to the one addressed directly to the establishments, contained questions on type and amount of activity on the two main arenas, the composition of various layers in the arenas and the maintenance regimens (Supplementary Material 2).

### Data Management

The arenas were classified into six main categories using questions on composition and maintenance. Synthetic arenas were deemed as arenas that contained rubber or waxed sand in the top-layer, irrespective of other categorisations. Grass arenas were classified directly by if they were deemed as grass (irrespective of other categorisations). Sand-fibre arenas were deemed as such if they were stated to contain fibre but were not wax or polymer. Sand-wood material arenas were classified as sand-woodchips when the top-layer or the reconstruction layers were stated as sand-woodchips or other types of biological forest material. Sand-mineral arenas were those that contained natural or manufactured sand (stone dust/stone crush), but were not deemed as any of the above categories. The last category was the unclassified arenas.

The studied arenas also had some features based on local tradition, i.e., the use of a layer of rubber or “Paddex” (further described in the discussion). For all arenas groupings were also made (irrespective of the above groupings) with respect to: a layer of rubber was deemed if any of layers 2–5 were declared as “rubber,” “Paddex” was deemed if any of layers 1–5 were declared as “Paddex” from the free-text answers and “Geotextile” was deemed to be present if any of the answers to this question was positive (for each layer of construction it was asked whether geotextile was included). Regarding desired property spans, if respondents answered that the desired property levels were above the provided upper limit of 5, these values were set to 5.

There were two questions relating to the respondents discipline. First the main discipline of the respondent, categorised as dressage, 3-day evening, show-jumping or other, was asked for. If respondents had answered “other” and provided a free-text answer that could be used for grouping, these answers were further grouped. These categorisations were all-round (if >2 disciplines provided or non-specific answers provided), driving, working equitation/western riding or gymkhana/vaulting/riding for disabled/monté-riding. In case only two alternatives were given to the free-text question and one of them was dressage, 3-day eventing, show-jumping, this alternative was selected for the general rider categorisation.

For most questions results have been shown as the questions were posed. Exceptions to this are most of the questions about composition of arenas that have primarily been used for the above arena classification and two free-text questions (additional opinions about surface characteristics and additional opinions about the whole questionnaire). For technical reasons a number of questions were posed as free-text questions (e.g., many questions related to the composition) and those were edited to be able to compare in categories or as continuous variables. Most arena questions have been contrasted against top-layer composition, for some questions separately for indoor and outdoor arenas. For some questions (was a company hired for the construction, is there a person allocated as responsible for the arena, is manure removed from the arena, frequency of renovation of the top-layer, economical lifespan, and yearly expected cost of maintenance) the answers have also been contrasted vs. usage frequency. To study the frequency of maintenance over all, dragging, harrowing, deep harrowing were combined, and contrasted to usage frequency.

### Statistics

Means, standard deviations, minima, maxima, and medians, or boxplots, have been used to demonstrate distributions for the continuous variables. Categorical variables have been shown as frequency distributions. To enable statistical comparisons 95% confidence intervals have been included (95% CI). For categorical variables exact confidence intervals have been used. Surface properties have been related to main discipline of the respondent (only the groups dressage, show-jumping, and eventing were included) and were analysed by the Kruskall-Wallis test, followed by two-way comparisons if the general comparison was significant. The watering frequency of the arenas over the seasons was analysed likewise, first Kruskall-Wallis was used for a general comparison between arena types within season after which pair-wise comparison was undertaken. Paired comparison of lower and upper spans for desired arena characteristics, between training and competition arenas, was done within rider discipline (dressage, show-jumping, and eventing) using the Wilcoxon rank sum test. The *p*-value limit was <0.05.

## Results

### General Results

Out of 4,824 riders who opened the questionnaire 3,158 riders provided at least one answer with respect to desired surface characteristics. The overall response rate was 3,158 of a total of 15,356 questionnaires distributed or 21%. The disciplines identified by the 3,158 riders were: show-jumping 1,458 (46%), dressage 1,429 (45%), eventing 147 (5%), driving 56 (2%), all-round 21 (1%), endurance 12 (0.4%), and working equitation/western riding 12 (0.4%). In addition 17 riders (0.6%) claimed their main discipline was vaulting, gymkhanas, or riding for the disabled as well as six riders that indicated “other” but without specifying a specific discipline.

Filling in of the questionnaire sent to riding establishments was initiated in 161 cases. From the rider and establishment questionnaires there was information describing at least one arena in 322 and 130 completed questionnaires, respectively. In 110 and 94 questionnaires for rider and establishment questionnaires a second arena was also indicated. The total number of arenas reported was then 656 (322+130+110+94). The response rate for the establishments could be defined as 130 out of 891 questionnaires distributed or 15%. Data are found in Supplementary Material 3.

### Arena Data

#### Distribution Indoor/Outdoor, Composition of the Top-Layer, and Construction

Of the 656 arenas sand-mineral surfaces represented one third, sand-woodchips arenas one third, and sand-fibre arenas represented 14% of all surveyed arenas. Table 1 demonstrates how top-layer composition distributes on indoor/outdoor arena location. Within the synthetic (*n* = 50) group 19 arenas were stated to contain waxed sand. Geotextiles (Table 2) were used less often in arenas with sand-woodchips top-layers when compared to sand-fibre top-layers. The percentage with geotextile in the four most common top-layer categories varied from 14 to 29%. A rubber layer was found in 17 indoor arenas and 23 outdoor arenas. Such a rubber layer was added to two sand-minerals top-layers (1%), four sand-woodchips top-layers (2%), seven of the sand-fibre top-layers (8%), and 27 of the synthetic top-layers (54%). The mineral based “Paddex” was used in two indoor and five outdoor arenas. Paddex was added to six of the arenas in the sand-minerals top-layer category and in one case was used in a sand-woodchips top-layer.

**Table 1:** Arenas by indoor/outdoor and top-layer category, including numbers, proportions and their 95% confidence intervals (95%CI).

	**Indoor**			**Outdoor**			**Total**		
**Category**	***n***	**%**	**95% CI**	***n***	**%**	**95% CI**	***n***	**%**	**95% CI**
Sand-mineral	34	12	(08, 16)	186	50	(45, 55)	220	34	(30, 37)
Sand-woodchips	153	54	(48, 60)	67	18	(14, 22)	220	34	(30, 37)
Sand-fibre	63	22	(18, 28)	28	8	(05, 11)	91	14	(11, 17)
Synthetic	20	7	(04, 11)	30	8	(05, 11)	50	8	(06, 10)
Grass	0	0		15	4	(02, 07)	15	2	(01, 04)
Unclassified	13	5	(02, 08)	47	13	(09, 16)	60	9	(07, 12)
Total	283	43	(39, 47)	373	57	(53, 61)	656	100	

*Data represent 656 riding arenas identified in a questionnaire in Sweden 2014*.

**Table 2:** Construction of arenas.

		**Top-layer category**												
		**Sand-**		**Sand**		**Sand-**		**Synthetic**		**Grass**		**Un-**		**Total**
		**minerals**		**woodchips**		**fibre**						**classified**		
		***n***	**%**	***n***	**%**	***n***	**%**	***n***	**%**	***n***	**%**	***n***	**%**	***n*** **/%**
Was a company hired for the construction?	Yes	40	(19)	48	(22)	55	(62)	20	(40)	1	(8)	15	(34)	179
		(14, 25)		(17, 28)		(51, 72)		(26, 55)		(00, 36)		(20, 50)		(28)
	No	132	(62)	127	(58)	29	(33)	24	(48)	11	(85)	14	(32)	347
		(55, 69)		(51, 65)		(22, 42)		(34, 63)		(55, 98)		(19, 48)		(54)
	Don't know	41	(19)	43	(20)	5	(6)	6	(12)	1	(8)	15	(34)	111
		(14, 25)		(15, 26)		(02, 12)		(05, 24)		(00, 36)		(20, 50)		(17)
Was the ground prepared during the original construction?	Drainage	67	(32)	65	(30)	23	(27)	8	(16)	1	(11)	23	(43)	187
		(26, 38)		(24, 37)		(18, 37)		(07, 30)		(00, 48)		(29, 57)		(30)
	Excavation	108	(51)	100	(46)	53	(62)	31	(63)	4	(44)	17	(31)	313
		(44, 58)		(40, 53)		(19, 31)		(48, 77)		(14, 79)		(20, 46)		(50)
	Don't know	36	(17)	51	(24)	10	(12)	10	(20)	4	(44)	14	(26)	125
		(12, 23)		(18, 30)		(02, 08)		(10, 34)		(14, 79)		(15, 40)		(20)
Were sieve analysis used when ordering arena material?	Yes	30	(15)	32	(17)	22	(28)	7	(16)	0	(0)	10	(28)	101
		(11, 21)		(12, 23)		(18, 39)		(07, 30)		(00, 28)		(14, 45)		(18)
	No	89	(46)	74	(39)	23	(29)	17	(39)	9	(82)	11	(31)	222
		(39, 53)		(32, 46)		(19, 40)		(24, 55)		(48, 98)		(16, 48)		(40)
	Don't know	76	(39)	86	(45)	34	(43)	20	(45)	2	(18)	15	(42)	233
		(32, 46)		(38, 52)		(32, 55)		(30, 61)		(02, 52)		(26, 59)		(42)
Is there a person allocated as responsible for the riding arena?	Yes	156	(74)	176	(88)	75	(89)	36	(84)	11	(85)	43	(86)	496
		(67, 80)		(83, 93)		(81, 95)		(69, 93)		(02, 45)		(18, 45)		(83)
	No	55	(26)	23	(12)	9	(11)	7	(16)	2	(15)	7	(14)	103
		(20, 33)		(07, 17)		(05, 19)		(07, 31)		(02, 45)		(06, 27)		(17)
Geotextile^a^ present?	Yes	46	(21)	31	(14)	25	(26)	12	(29)	0	(0)	3	(5)	117
		(16, 27)		(10, 19)		(20, 39)		(12, 40)		(00, 22)		(01, 13)		(18)
Total *n* per arena type		223		220		95		41		15		62		656

*Numbers, percentages, and 95% confidence intervals (row below), per top-layer category from answers with information on each questions. Data are from 656 riding arenas from a questionnaire in Sweden 2014*.

a *The percentage for whether geotextiles is present is of all 656, while the denominator in the other questions are the total that answered the question*.

The age of the arenas were from oldest to youngest; sand-woodchips, sand-minerals, and sand-fibre (with non-overlapping 95% CIs, Table 3). The synthetic arenas were, in general, more than 15 years old. The synthetic arenas were shorter, but just as wide, with an area that was not smaller than the other four most common top-layer categories. The small number of grass arenas, *n* = 12 arenas which included data on the size, were generally wider and with a larger area but not always longer. The mean depth of the top-layer was similar for the sand-minerals, sand-woodchips, sand-fibre, and synthetic arenas. There were less data provided on the depth of the second layer but the depths were similar within this layer (Table 3).

**Table 3:** Data on age, size, and layer depth in arenas.

			***N***	**Mean**	**SD**	**Min**	**Median**	**Max**	**95% CI**
When founded?	Year	Sand-minerals	207	2002.6	9.0	1,973	2,005	2,014	(2,001, 2,004)
		Sand wood-c^a^	201	1998.7	12.2	1,945	2,000	2,014	(1,997, 2,000)
		Sand-fibre	93	2006.8	8.3	1,974	2,010	2,014	(2,005, 2,008)
		Synthetic	41	2002.7	10.1	1,977	2,007	2,014	(2,000, 2,006)
		Grass	12	2003.0	15.5	1,960	2,010	2,014	(1,994, 2,012)
		Unclassified	50	2003.7	9.0	1,980	2005.5	2,015	(2,001, 2,006)
Size	Length (m)	Sand-minerals	220	58.5	17.3	22	60	130	(56, 61)
		Sand wood-c	216	56.0	14.4	17	60	100	(54, 58)
		Sand-fibre	94	60.3	11.6	30	60	82	(58, 63)
		Synthetic	41	54.5	11.3	38	60	77	(51, 58)
		Grass	12	198.8	330.0	35	85	1200	(12, 385)
		Unclassified	56	61.7	16.4	22	60	100	(57, 66)
	Width (m)	Sand-minerals	220	29.6	15.7	6	24	100	(28, 32)
		Sand wood-c	215	25.7	12.5	10	22	100	(24, 27)
		Sand-fibre	94	24.4	6.9	17	22	50	(23, 26)
		Synthetic	41	24.1	6.4	18	22	50	(22, 26)
		Grass	12	60.3	53.7	5	45	200	(30, 91)
		Unclassified	55	31.6	15.5	20	25	80	(27, 36)
	Area (m2)	Sand-minerals	220	1,885	1,682	240	1,372	13,000	(1,663, 2,107)
		Sand wood-c	94	1,500	602	510	1,320	3,362	(1,378, 1,622)
		Sand-fibre	94	1,500	602	510	1,320	3,362	(1,378, 1,622)
		Synthetic	41	1,329	480	684	1,200	2,600	(1,182, 1,476)
		Grass	12	11,117	22,079	525	4,450	80,000	–(1,375, 23,610)
		Unclassified	55	2,075	1,541	800	1,500	8,000	(1,668, 2,482)
Top-layer	Depth (cm)	Sand-minerals	151	10.7	6.4	2	10	35	(10, 12)
		Sand wood-c	165	11.0	6.9	1	10	50	(10, 12)
		Sand-fibre	73	10.6	4.3	1	10	30	(10, 12)
		Synthetic	31	8.6	4.6	2.5	7	18	(07, 10)
		Grass	Not Applicable						
		Unclassified	26	10.3	6.5	3	9	30	(08, 13)
Second layer	Depth (cm)	Sand-minerals	70	12.0	8.7	0	10	50	(10, 14)
		Sand wood-c	78	12.2	8.9	0.5	10	50	(10, 14)
		Sand-fibre	43	14.8	11.2	3	10	65	(11, 18)
		Synthetic	15	12.2	7.8	3	10	30	(08, 16)
		Grass	Not Applicable						
		Unclassified	13	13.5	9.1	3	12	30	(09, 19)

*Distributions per top-layer category, including 95% confidence intervals (95% CIs), data from 656 riding arenas identified in a questionnaire in Sweden 2014*.

a *Sand wood-c, sand-woodchips*.

A construction firm was often hired (62%) when building a sand-fibre arena, while they were seldom used when constructing arenas with other top-layer materials (Table 2). About a fourth of all arenas had a drainage layer included in the construction, while well over 50% had the native soil excavated during construction (Table 2). For the four most common arena top-layers between 15 and 28% reported the use of sieve curves to specify the sand used. Overall though 39–45% reported not knowing whether sieve curves were used. Of all arenas, 86% had a person who was assigned responsibility for maintenance of the arena.

#### Practical Maintenance

Comparing the three most common arenas types, dragging was most frequently performed on sand-woodchips and sand-fibre arenas (Tables 4, 5). Harrowing was used less often on sand-mineral arenas compared to sand-woodchips and sand-fibre arenas. For deep harrowing the statistics do not show a large differences between the top-layers, likely because deep harrowing is generally done infrequently. Looking at the numbers (not the confidence intervals) rolling was more common on sand-fibre top-layers. Combining dragging, harrowing, deep harrowing and rolling, the figures in Table 6 suggest that arenas with higher usage were maintained more frequently.

**Table 4:** Frequency distributions of means used to maintain the arenas.

**Frequency per week**		**Sand-minerals**		**Sand-woodchips**		**Sand-fibre**		**Synthetic**		**Grass**		**Unclassified**		**Total**
		***n***	**%**	***n***	**%**	***n***	**%**	***n***	**%**	***n***	**%**	***n***	**%**	***n*** **/%**
Dragging	Daily	10	(4)	26	(16)	18	(27)	4	(11)	0	(0)	4	(9)	62
		(03, 10)		(11, 23)		(17, 39)		(03, 25)		(00, 71)		(02, 21)		(13)
	4–6	14	(8)	21	(13)	12	(18)	6	(16)	0	(0)	3	(7)	56
		(04, 13)		(08, 19)		(10, 29)		(06, 32)		(00, 71)		(01, 18)		(11)
	2–3	37	(21)	32	(20)	16	(24)	10	(27)	0	(0)	13	(29)	108
		(15, 27)		(14, 27)		(14, 36)		(14, 44)		(00, 71)		(16, 44)		(22)
	Once	66	(37)	43	(27)	12	(18)	12	(32)	0	(0)	17	(38)	150
		(30, 45)		(20, 35)		(10, 29)		(18, 50)		(00, 71)		(24, 53)		(31)
	Not so often	51	(29)	37	(23)	9	(13)	5	(14)	3	(100)	8	(18)	113
		(22, 36)		(17, 31)		(06, 24)		(05, 29)		(29, 100)		(08, 32)		(23)
Harrowing	Daily	7	(4)	21	(11)	11	(16)	7	(19)	0	(0)	2	(9)	48
		(02, 09)		(07, 16)		(08, 26)		(08, 35)		(00, 09)		(01, 29)		(9)
	4–6	9	(5)	39	(20)	16	(23)	0	(0)	0	(0)	1	(2)	65
		(03, 10)		(15, 26)		(14, 34)		(00, 09)		(00, 09)		(00, 12)		(13)
	1–3	20	(12)	45	(23)	16	(23)	10	(27)	0	(0)	6	(13)	97
		(08, 18)		(17, 30)		(14, 34)		(14, 44)		(00, 09)		(05, 27)		(19)
	Once	52	(32)	44	(23)	13	(19)	10	(27)	0	(0)	11	(24)	130
		(25, 39)		(17, 29)		(10, 30)		(14, 44)		(00, 09)		(13, 40)		(26)
	Not so often	77	(47)	45	(23)	14	(20)	10	(27)	3	(8)	18	(40)	167
		(39, 55)		(17, 30)		(11, 31)		(14, 44)		(02, 21)		(26, 56)		(33)
Deep harrowing	Daily	0	(0)	1	(1)	1	(3)	0	(0)	0	(0)	0	(18)	2
		(00, 03)		(00, 03)		(00, 14)		(00, 18)		(00, 60)		(05, 40)		(1)
	4–6	0	(0)	1	(1)	1	(3)	0	(0)	0	(0)	0	(14)	2
		(00, 03)		(00, 03)		(00, 14)		(00, 18)		(00, 60)		(03, 35)		(1)
	1–3	5	(4)	10	(6)	2	(5)	0	(0)	0	(0)	0	(59)	17
		(01, 10)		(03, 11)		(01, 18)		(00, 18)		(00, 60)		(36, 79)		(05,
	Once	14	(12)	29	(18)	5	(13)	0	(0)	1	(25)	0	(77)	49
		(07, 20)		(13, 25)		(04, 28)		(00, 18)		(01, 81)		(55, 92)		(14)
	Not so often	94	(83)	117	(74)	29	(76)	19	###	3	(75)	22	(36)	284
		(75, 90)		(66, 81)		(60, 89)		(82, 100)		(19, 99)		(17, 59)		(80)
Rolling	Daily	0	(0)	0	(0)	3	(7)	0	(0)	0	(0)	0	(0)	3
		(00, 05)		(00, 05)		(01, 18)		(00, 18)		(00, 60)		(00, 26)		(1)
	4–6	0	(0)	0	(0)	2	(4)	0	(0)	0	(0)	0	(0)	2
		(00, 05)		(00, 05)		(01, 15)		(00, 18)		(00, 60)		(00, 26)		(1)
	1–3	4	(5)	7	(9)	12	(27)	2	(11)	0	(0)	0	(0)	25
		(01, 13)		(04, 17)		(15, 42)		(01, 33)		(00, 60)		(00, 26)		(11)
	Once	1	(1)	4	(5)	2	(4)	7	(37)	0	(0)	1	(8)	15
		(00, 07)		(01, 12)		(01, 15)		(16, 62)		(00, 60)		(00, 38)		(6)
	Not so often	73	(94)	69	(86)	26	(58)	10	(53)	4	(100)	11	(92)	193
		(86, 98)		(77, 93)		(42, 72)		(29, 76)		(40, 100)		(62, 100)		(81)
Total *n* per arena type	223		220		95		41		15		62		656

*Percentages, and 95% confidence intervals (row below), per top-layer category from answers with information on each questions. Data are from 656 riding arenas from a questionnaire in Sweden 2014*.

**Table 5:** Frequency distributions of means used to maintain the arenas.

**Question**		**Sand-minerals**		**Sand-woodchips**		**Sand-fibre**		**Synthetic**		**Grass**		**Unclassified**		**Total**
How often is watering undertaken? (per week)	Daily	5	(4)	14	(8)	15	(19)	7	(18)	0	(0)	2	(7)	43
		(01, 08)		(04, 13)		(11, 30)		(08, 34)		(00, 52)		(01, 24)		(9)
	4–6	7	(5)	21	(12)	11	(14)	4	(10)	1	(20)	0	(0)	44
		(02, 10)		(08, 18)		(07, 24)		(03, 24)		(01, 72)		(00, 12)		(10)
	1–3	16	(12)	49	(28)	13	(17)	3	(8)	1	(20)	3	(11)	85
		(07, 18)		(21, 35)		(09, 27)		(02, 21)		(01, 72)		(02, 28)		(18)
	Once	22	(16)	45	(26)	17	(22)	5	(13)	0	(0)	3	(11)	92
		(10, 23)		(19, 33)		(13, 33)		(04, 27)		(00, 52)		(02, 28)		(20)
	Not so often	86	(63)	47	(27)	22	(28)	20	(51)	3	(60)	20	(71)	198
	Often	(55, 71)		(20, 34)		(19, 40)		(35, 68)		(15, 95)		(51, 87)		(43)
How often is other maintenance undertaken? (per week)	Daily	1	(2)	3	(12)	0	(0)	1	(8)	0	(0)	0	(0)	5
		(00, 11)		(02, 30)		(00, 34)		(00, 36)		(00, 34)		(00, 31)		(4)
	4–6	0	(0)	1	(4)	0	(0)	1	(8)	0	(0)	0	(0)	2
		(00, 00)		(00, 20)		(00, 34)		(00, 36)		(00, 34)		(00, 31)		(2)
	1–3	2	(4)	0	(0)	0	(0)	0	(0)	0	(0)	2	(20)	4
		(01, 14)		(00, 13)		(00, 34)		(00, 25)		(00, 34)		(03, 56)		(3)
	Once	0	(0)	1	(4)	1	(11)	2	(15)	3	(33)	0	(0)	7
		(00, 07)		(00, 13)		(00, 34)		(00, 25)		(00, 34)		(03, 56)		(6)
	Not so often	45	(94)	21	(81)	8	(89)	9	(69)	6	(67)	8	(80)	97
	Often	(83, 99)		(61, 93)		(52, 100)		(39, 91)		(30, 93)		(44, 97)		(84)
Is there a sprinkler system for watering?	Yes	25	(12)	53	(25)	39	(45)	11	(23)	2	(14)	13	(24)	143
		(08, 17)		(19, 32)		(35, 56)		(12, 37)		(02, 43)		(13, 38)		(23)
	No	183	(88)	158	(75)	47	(55)	37	(77)	12	(86)	41	(76)	478
		(83, 92)		(68, 81)		(44, 65)		(63, 88)		(57, 98)		(62, 87)		(77)
Is a watertank used for watering?	Yes	44	(22)	51	(25)	21	(25)	8	(18)	0	(0)	9	(19)	133
		(16, 28)		(19, 32)		(16, 35)		(08, 33)		(00, 26)		(09, 33)		(22)
	No	158	(78)	152	(75)	64	(75)	36	(82)	12	(100)	38	(81)	460
		(72, 84)		(68, 81)		(65, 84)		(67, 92)		(74, 100)		(67, 91)		(78)
Is the arena watered by hose?	Yes	116	(60)	120	(64)	42	(53)	25	(54)	5	(38)	17	(41)	325
		(53, 67)		(57, 71)		(41, 64)		(39, 69)		(14, 68)		(26, 58)		(58)
	No	78	(40)	67	(36)	38	(48)	21	(46)	8	(62)	24	(59)	236
		(33, 47)		(29, 43)		(36, 59)		(31, 61)		(32, 86)		(42, 74)		(42)
Is manure removed from the arena?	Yes	189	(88)	193	(91)	89	(99)	43	(93)	9	(69)	45	(88)	568
		(83, 92)		(86, 94)		(94, 100)		(82, 99)		(39, 91)		(76, 96)		(90)
	No	26	(12)	20	(9)	1	(1)	3	(7)	4	(31)	6	(12)	60
		(08, 17)		(06, 14)		(00, 06)		(01, 18)		(09, 61)		(04, 24)		(10)
Is the arena salted?	Yes	78	(37)	144	(68)	44	(50)	25	(53)	1	(8)	20	(40)	312
		(30, 43)		(61, 74)		(39, 61)		(38, 68)		(00, 36)		(26, 55)		(50)
	No	135	(63)	68	(32)	44	(50)	22	(47)	12	(92)	30	(60)	311
		(57, 70)		(26, 39)		(39, 61)		(32, 62)		(64, 100)		(45, 74)		(50)
Total *n* per arena type		223		220		95		41		15		62		656

*Percentages, and 95% confidence intervals (row below), are per top-layer category from answers with information on each questions. Data are from 656 riding arenas from a questionnaire in Sweden 2014*.

*^a^The question about watering is here shown as posed, while in Figures 3A,B shows watering by season and in numerical categories constructed from the data*.

**Table 6:** Combined maintenance regimes contrasted to usage frequency and the frequency of watering contrasted to usage frequency.

		**Sessions per day**								
		**1–10**		**11–20**		**21–40**		** <40**		**Total**
		***n***	**%**	***n***	**%**	***n***	**%**	***n***	**%**	***n*** **/%**
How often is maintenance undertaken? (times per week)	Daily	22	(7)	10	(16)	19	(19)	35	(36)	86
		(04, 10)		(08, 27)		(12, 28)		(26, 46)		(13)
	4–6	28	(8)	18	(28)	25	(25)	19	(19)	90
		(06, 12)		(18, 41)		(17, 35)		(12, 29)		(11)
	2–3	75	(23)	29	(45)	25	(25)	26	(27)	155
		(18, 28)		(33, 58)		(17, 35)		(18, 36)		(22)
	Once a week	137	(41)	21	(33)	17	(17)	11	(11)	186
		(36, 47)		(22, 46)		(10, 26)		(06, 19)		(31)
	Not so often	69	(21)	4	(6)	14	(14)	7	(7)	113
		(17, 26)		(02, 15)		(08, 22)		(03, 14)		(23)
How often is watering undertaken? (times per week)	Daily	18	(8)	6	(10)	9	(10)	10	(12)	43
		(05, 12)		(04, 21)		(05, 19)		(06, 21)		(9)
	4–6	11	(5)	5	(8)	8	(9)	20	(24)	44
		(02, 09)		(03, 18)		(04, 18)		(16, 35)		(10)
	2–3	29	(13)	15	(25)	21	(24)	20	(24)	85
		(09, 18)		(15, 38)		(16, 35)		(16, 35)		(18)
	Once a week	41	(18)	14	(23)	21	(24)	15	(18)	92
		(13, 24)		(13, 36)		(16, 35)		(11, 28)		(20)
	Not so often	127	(56)	25	(42)	27	(31)	17	(21)	198
		(49, 63)		(29, 55)		(22, 42)		(13, 31)		(43)

*Percentage figures, and 95% confidence intervals, shown below the number n and the percentage are per usage category, defined as sessions per day. Data are from 656 riding arenas from a questionnaire in Sweden 2014*.

#### Long-Term Plans for Maintenance

Considering the top-layers with a reasonably high number of responses, top-layer renovation was most commonly planned for every other-fourth year or every fifth to 10th year (Table 7). Arenas with a sand-woodchips top-layer were, in general, associated with the shortest duration between required renovations. Few respondents allocated more than 10,000 SEK in yearly maintenance costs, with the exception of sand-woodchips and sand-fibre arenas followed by synthetic arenas. In total 33 respondents said they had a plan for disposing of their arena material when used and 175 did not (8 vs. 41%, based on 432 respondents answering once each). When the usage frequency is considered along with the arena top-layer no clear pattern emerged. For example synthetic or sand-fibre arenas did not have more frequent disposal plans than, for example, sand-woodchips arenas (data not shown).

**Table 7:** Plans for long-term maintenance of the arenas.

		**Sand-minerals**		**Sand-woodchips**		**Sand-fibre**		**Synthetic**		**Grass**		**Unclassified**		**Total**
		***n***	**%**	***n***	**%**	***n***	**%**	***n***	**%**	***n***	**%**	***n***	**%**	***n*** **/%**
How often do you think the top-layer should be renovated? (per years)	Once a year a year	31	(15)	57	(27)	20	(23)	3	(7)	4	(33)	7	(16)	122
		(11, 21)		(21, 33)		(14, 33)		(01, 19)		(10, 65)		(06, 29)		(20)
	Every other-4th year	76	(37)	98	(46)	32	(36)	13	(30)	2	(17)	15	(33)	236
		(31, 44)		(39, 53)		(26, 47)		(17, 46)		(02, 48)		(20, 49)		(39)
	Every 5th-10th year	64	(31)	49	(23)	27	(31)	17	(40)	2	(17)	19	(42)	178
		(25, 38)		(18, 29)		(21, 41)		(25, 56)		(02, 48)		(30, 60)		(29)
	More than every 10th year	33	(16)	8	(4)	9	(10)	10	(23)	4	(33)	4	(9)	68
		(11, 22)		(02, 07)		(05, 19)		(12, 39)		(10, 65)		(02, 21)		(11)
What ingredients are used for top-layer renovation?	Sand/minerals	108	(48)	80	(21)	40	(20)	14	(13)	2	(43)	16	(33)	260
		(42, 55)		(30, 43)		(32, 53)		(20, 51)		(02, 40)		(18, 42)		(40)
	Woodchips	0	(0)	124	(32)	0	(0)	0	(0)	0	(0)	0	(0)	124
		(00, 02)		(50, 63)		(00, 04)		(00, 09)		(00, 22)		(00, 06)		(19)
	Fibre	0	(0)	3	(1)	62	(31)	9	(9)	0	(0)	0	(0)	74
		(00, 02)		(00, 04)		(55, 75)		(11, 38)		(00, 22)		(00, 06)		(11)
	Rubber	0	(0)	2	(1)	0	(0)	18	(17)	0	(0)	0	(0)	20
		(00, 02)		(00, 03)		(00, 04)		(28, 60)		(00, 22)		(00, 06)		(3)
	Saw dust	0	(0)	50	(13)	0	(0)	0	(0)	0	(0)	0	(0)	50
		(00, 02)		(17, 29)		(00, 04)		(00, 09)		(00, 22)		(00, 06)		(8)
	Stone-meal	64	(29)	30	(8)	8	(4)	5	(5)	1	(21)	15	(30)	123
		(23, 35)		(09, 19)		(04, 16)		(04, 26)		(00, 32)		(16, 38)		(19)
What is the economic duration of the arena? (years)	1–3	7	(5)	18	(10)	2	(3)	2	(6)	0	(0)	3	(10)	32
		(02, 10)		(06, 15)		(00, 09)		(01, 20)		(00, 71)		(02, 27)		(7)
	4–6	27	(19)	52	(21)	8	(11)	1	(3)	0	(0)	5	(17)	93
		(13, 26)		(22, 35)		(05, 20)		(00, 16)		(00, 71)		(06, 36)		(20)
	7–9	18	(13)	41	(22)	17	(22)	5	(15)	1	(33)	7	(24)	89
		(08, 19)		(16, 29)		(14, 33)		(05, 32)		(01, 91)		(15, 51)		(19)
	10–12	29	(21)	41	(22)	20	(26)	9	(27)	0	(0)	5	(17)	104
		(14, 28)		(16, 29)		(17, 38)		(13, 46)		(00, 71)		(06, 36)		(22)
	13–15	8	(6)	12	(6)	8	(11)	7	(21)	0	(0)	4	(14)	39
		(02, 11)		(03, 11)		(05, 20)		(09, 39)		(00, 71)		(04, 32)		(8)
	Over 15	52	(37)	22	(12)	21	(28)	9	(27)	2	(67)	5	(17)	111
		(29, 45)		(08, 17)		(18, 39)		(13, 46)		(25, 99)		(06, 36)		(24)
What is the early expected cost for the arena? (SEK^a^)	0–1,000	71	(38)	24	(12)	13	(17)	13	(34)	7	(54)	12	(34)	140
		(31, 45)		(08, 18)		(09, 27)		(20, 51)		(25, 81)		(21, 55)		(26)
	1,000–5,000	56	(30)	53	(27)	15	(19)	9	(24)	4	(31)	11	(31)	148
		(23, 37)		(21, 34)		(11, 30)		(11, 40)		(09, 61)		(17, 49)		(27)
	5,000–10,000	38	(20)	57	(30)	16	(21)	6	(16)	2	(15)	4	(11)	123
		(15, 26)		(23, 37)		(12, 32)		(06, 31)		(02, 45)		(05, 30)		(23)
	10,000–20,000	8	(4)	32	(17)	14	(18)	5	(13)	0	(0)	5	(14)	64
		(02, 08)		(12, 23)		(10, 29)		(04, 28)		(00, 25)		(05, 30)		(12)
	20,000–30,000	6	(3)	20	(10)	5	(6)	4	(11)	0	(0)	1	(3)	36
		(01, 07)		(06, 16)		(02, 25)		(03, 25)		(00, 25)		(00, 15)		(7)
	30,000–40,000	5	(3)	6	(3)	5	(6)	0	(0)	0	(0)	1	(3)	17
		(01, 06)		(01, 07)		(02, 15)		(00, 09)		(00, 25)		(00, 15)		(3)
	40,000–50,000	2	(1)	0	(0)	1	(1)	0	(0)	0	(0)	1	(3)	4
		(00, 04)		(00, 02)		(00, 07)		(00, 09)		(00, 25)		(00, 15)		(1)
	> 50,000	3	(2)	1	(1)	8	(10)	1	(3)	0	(0)	0	(0)	14
		(00, 05)		(00, 03)		(05, 19)		(00, 14)		(00, 25)		(00, 10)		(3)
Total *n* per arena type	223		220		95		41		15		62		656

*Percentage figures, and 95% confidence intervals, shown below the number (n) and the percentage are per arena type. The denominator for the ingredients for top-layer renovation (2nd question) is all of a specific top-layer (as several answers are possible)*.

a *One dollar corresponded to 6.9 SEK in 2014(https://www.exchangerates.org.uk/USD-SEK-spot-exchange-rates-history-2014.html)*.

#### Arenas Usage

**Figure 1:**
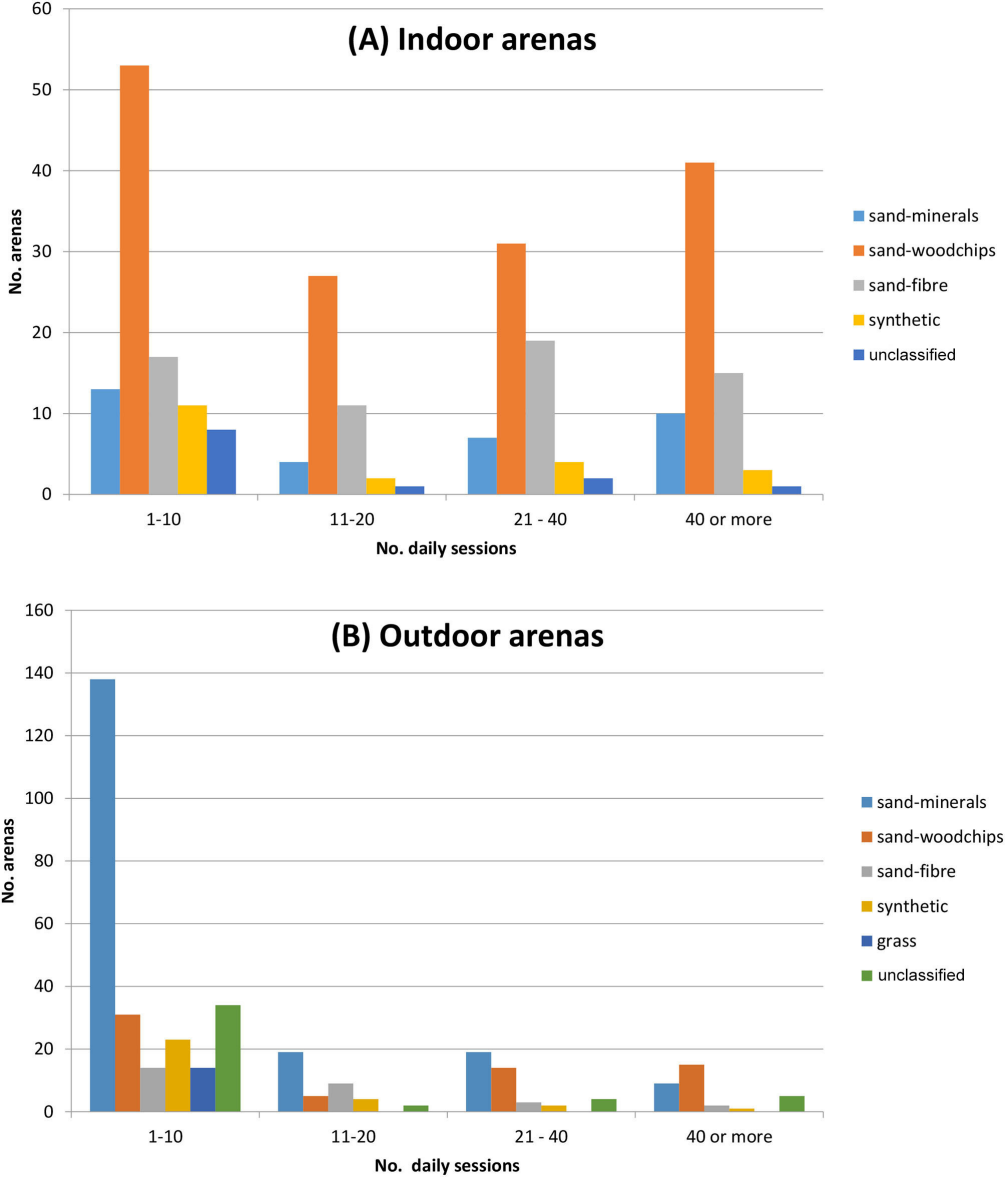
Daily sessions per top-layer in indoor **(A)** and outdoor **(B)** arenas. Number of arenas per top-layer category and number of daily sessions (a session defined as 45–60 min work from one horse). Data are from 280 indoor and 367 outdoor riding arenas identified in a questionnaire in Sweden 2014.

**Figure 2:**
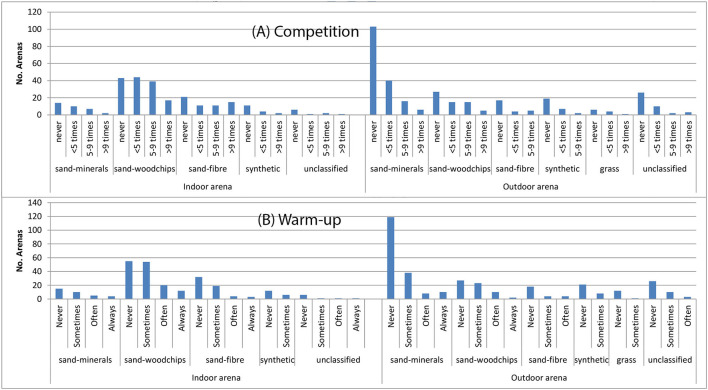
Number of times arenas are used **(A)** for competition per year and **(B)** as warm-up for competition. Data from a pool of 656 riding arenas identified in a questionnaire in Sweden 2014.

Table 8 shows that dressage and show-jumping were the main disciplines in the population of arenas, i.e., by far the most common. Compared to the indoor arenas, the outdoor arenas were commonly used less often (the blue bars are large and other bars are smaller) (Figures 1A,B). Figure 2 demonstrates the usage in competition (times per year), and warm-up for competition, for the different arena types and by indoor/outdoor arenas. Indoor sand-woodchips arenas were most commonly used in competition. A relatively large number of sand-minerals, sand-fibre, and synthetic top-layers arenas were never used for warm-up.

**Table 8:** The disciplines performed on the arenas.

**Rank**		**Dressage**	**Driving**	**Endurance**	**Eventing**	**Gymkhana**	**Icelandic**	**Reining**	**Show-**	**Western**	**Vaulting**	**Working**
									**jumping**			**equitation**
1	*n*	251	8	0	8	2	1	0	124	0	4	1
	(%)	(38)	(1)	(0)	(1)	(0)	(0)	(0)	(19)	0	(1)	(0)
2	*n*	96	4	2	8	0	3	3	179	2	3	8
	(%)	(15)	(1)	(0)	(1)	(0)	(0)	(0)	(27)	(0)	(0)	(1)
3	*n*	21	34	3	34	5	11	0	19	15	7	16
	(%)	(3)	(5)	(0)	(5)	(1)	(2)	(0)	(3)	(2)	(1)	(2)
4	*n*	5	14	1	11	9	8	2	7	14	11	10
	(%)	(1)	(2)	(0)	(2)	(1)	(1)	(0)	(1)	(2)	(2)	(2)
5	*n*	3	10	3	7	5	5	3	9	6	10	1
	(%)	(0)	(2)	(0)	(1)	(1)	(1)	(0)	(1)	(1)	(2)	(0)
6	*n*	0	9	2	4	4	4	4	9	7	3	6
	(%)	(0)	(1)	(0)	(1)	(1)	(1)	(1)	(1)	(1)	(0)	(1)
7	*n*	2	1	2	5	9	4	3	3	3	9	5
	(%)	(0)	(0)	(0)	(1)	(1)	(1)	(0)	(0)	(0)	(1)	(1)
8	*n*	2	5	1	1	7	4	3	4	6	4	4
	(%)	(0)	(1)	(0)	(0)	(1)	(1)	(0)	(1)	(1)	(1)	(1)
9	*n*	3	1	6	3	3	3	6	6	4	4	8
	(%)	(0)	(0)	(1)	(0)	(0)	(0)	(1)	(1)	(1)	(1)	(1)
10	*n*	3	5	2	4	6	7	6	6	2	3	4
	(%)	(0)	(1)	(0)	(1)	(1)	(1)	(1)	(1)	(0)	(0)	(1)
11	*n*	4	3	6	1	4	5	3	3	1	4	4
	(%)	(1)	(0)	(1)	(0)	(1)	(1)	(0)	(0)	(0)	(1)	(1)
12	*n*	10	2	8	6	0	3	0	2	3	5	3
	(%)	(2)	(0)	(1)	(1)	(0)	(0)	(0)	(0)	(0)	(1)	(0)
	*n*	400	96	36	92	54	58	33	371	63	67	70
	(%)	(61)	(15)	(5)	(14)	(8)	(9)	(5)	(57)	(10)	(10)	(11)

*They are ranked from 1- the most often performed discipline to 12- the least often performed discipline. The percentages represent the proportion of all 656 arenas*.

#### Watering

Table 5 shows the frequency of watering per week, the presence of a sprinkler system and whether a water tank and/or a hose was used for watering. Of the arenas, 143 had water sprinkler systems. Comparing to all arenas, sprinkler systems was found in 30 and 16% of the indoor/outdoor arenas, respectively. Sand-fibre arenas had sprinkler systems more often when compared to sand-woodchips arenas and sand-woodchips arenas had sprinkler systems more often than sand-mineral arenas (Table 5). Sand-mineral arenas were also salted less often than sand-woodchips arenas (Table 5).

**Figure 3:**
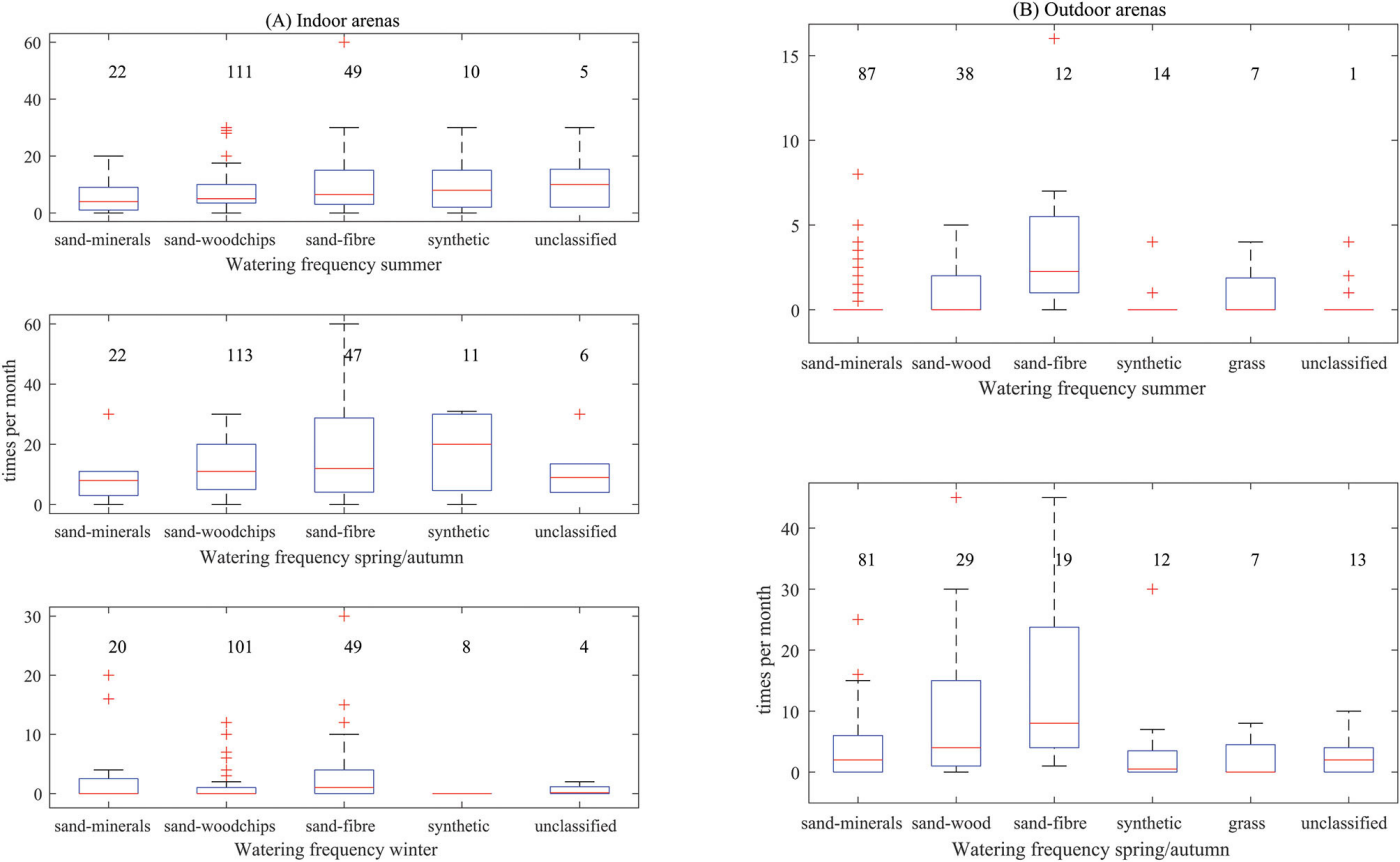
Watering frequency by season for indoor **(A)** and outdoor **(B)** arenas by top-layer category. Data from a pool of 656 riding arenas identified in a questionnaire in Sweden 2014. N for each category is given in the graph.

For watering of indoor arenas, the different arena top-layers only showed group-level statistical differences during the winter (Figure 3A, *p* = 0.006). The arenas with a sand-woodchips top-layer were watered less (1.1 times per month, standard deviation of 1.0) than the sand-fibre top-layer arenas (3.6 times per month, standard deviation of 6.7, *p* = 0.002). Sand-fibre arenas were also watered more frequently than the synthetic areas which were not watered at all (*p* = 0.006).

During summer (Figure 3B) there was a statistical difference for outdoor arenas (group-level *p* = 0.0004). Arenas with a sand-woodchips top-layer were watered more frequently (1.6 times per month, standard deviation of 1.7) than sand-mineral areas (0.7 times per month, standard deviation of 1.5, *p* = 0.03). Arenas with a sand-fibre top-layer were watered more frequently (3.7 times per month, standard deviation of 4.5), than sand-minerals (*p* < 0.0001), sand-woodchips (1.2 times per month, standard deviation of 1.7, *p* = 0.02), synthetic (0.4 times per month, standard deviation of 1.1, *p* = 0.002) and unclassified (0.4 times per month standard deviation of 1.1, *p* = 0.001).

In spring/autumn there was also a statistical difference for the outdoor arena top-layers (group-level *p* = 0.002). Sand-fibre top-layers (12.7 times per month, standard deviation of 12.9) were watered more frequently than sand-minerals (3.9 times per month, standard deviation of 4.6, *p* = 0.001), synthetic (3.9 times per month, standard deviation of 8.5, *p* = 0.006), grass surfaces (2.3 times per month standard deviation of 3.2, *p* = 0.01) and unclassified (2.7 times per month, standard deviation of 2.8, *p* = 0.008).

#### Some Specific Questions Related to Frequency of Usage

Arenas with more frequent usage were more frequently initially constructed by a company. It was also expected that more frequent renovations would be required for more heavily used arenas (e.g., yearly renewal comparing 1–10 and <40 sessions per day). With high usage, the lifespan of the arenas would be shorter and the expected cost of maintenance higher (Table 9). Manure removal and the existence of a designated person for maintenance did not differ between usage categories. The perception of the need for arena renewal was associated with the number of daily sessions. Arenas with high expectations for maintenance cost were those arenas used most frequently. The figures in Table 6 suggest that arenas with higher usage were also maintained and watered more frequently (some confidence intervals are non-overlapping).

**Table 9:** Selected questions contrasted to questions on usage frequency.

		**Sessions per day**								
		**1–10**		**11–20**		**21–40**		**>40**		**Total**
		***n***	**%**	***n***	**%**	***n***	**%**	***n***	**%**	***n*** **/%**
Was a company hired for the construction?	Yes	83	(24)	27	(33)	27	(26)	41	(40)	178
		(20, 29)		(23, 44)		(18, 36)		(31, 50)		(28)
	No	212	(61)	39	(48)	55	(54)	39	(38)	345
		(56, 66)		(36, 59)		(44, 64)		(29, 48)		(55)
	Don't know	52	(15)	16	(20)	20	(20)	22	(22)	110
		(11, 19)		(12, 30)		(12, 29)		(14, 31)		(17)
Is there a person allocated as responsible for the riding arena?	Yes	260	(81)	68	(85)	81	(83)	83	(86)	492
		(76, 85)		(75, 92)		(74, 90)		(78, 93)		(83)
	No	61	(19)	12	(15)	17	(17)	13	(14)	103
		(15, 24)		(08, 25)		(10, 26)		(07, 22)		(17)
Is manure removed from the arena?	Yes	314	(91)	74	(93)	87	(86)	89	(90)	564
		(88, 94)		(84, 97)		(78, 92)		(82, 95)		(90)
	No	30	(9)	6	(8)	14	(14)	10	(10)	60
		(06, 12)		(03, 16)		(08, 22)		(05, 18)		(10)
How often do you think the top-layer should be renovated?	Once a year (yr)	44	(13)	8	(10)	27	(28)	42	(42)	121
		(10, 18)		(05, 19)		(19, 37)		(33, 53)		(20)
	Every other-4th yr	121	(37)	31	(40)	38	(39)	44	(44)	234
		(32, 43)		(29, 52)		(29, 49)		(34, 55)		(39)
	Every 5th-10th yr	110	(34)	29	(38)	27	(28)	11	(11)	177
		(29, 39)		(27, 49)		(19, 37)		(06, 19)		(30)
	Less than every 10th yr	51	(16)	9	(12)	6	(6)	2	(2)	68
		(12, 20)		(05, 21)		(02, 13)		(00, 07)		(11)
What is the economic lifespan of the arena?	1–3 yrs	10	(4)	2	(4)	6	(8)	14	(16)	32
		(02, 07)		(00, 13)		(03, 17)		(09, 26)		(7)
	4–6 yrs	29	(11)	10	(20)	18	(24)	35	(41)	92
		(08, 16)		(10, 33)		(15, 35)		(30, 52)		(20)
	7–9 yrs	46	(18)	11	(22)	22	(29)	10	(12)	89
		(14, 23)		(11, 35)		(19, 41)		(06, 20)		(19)
	10–12 yrs	56	(22)	12	(24)	14	(19)	21	(24)	103
		(17, 28)		(13, 37)		(11, 29)		(16, 35)		(22)
	13–15 yrs	29	(11)	7	(14)	1	(1)	2	(2)	39
		(08, 16)		(06, 26)		(00, 07)		(00, 08)		(8)
	Over 15 yrs	84	(33)	9	(18)	14	(19)	4	(5)	111
		(27, 39)		(08, 31)		(11, 29)		(01, 11)		(24)
What is the yearly expected cost for the arena surface (SEK^a^)?	0–1,000	105	(34)	8	(14)	25	(30)	2	(2)	140
		(29, 40)		(06, 25)		(21, 41)		(00, 08)		(26)
	1,000–5,000	106	(34)	17	(29)	10	(12)	15	(16)	148
		(29, 40)		(18, 42)		(06, 21)		(09, 25)		(27)
	5,000–10,000	63	(20)	18	(31)	26	(31)	13	(14)	120
		(16, 25)		(19, 44)		(22, 42)		(08, 23)		(22)
	10,000–20,000	22	(7)	13	(22)	12	(14)	17	(18)	64
		(05, 11)		(12, 35)		(08, 24)		(11, 28)		(12)
	20,000–30,000	7	(2)	2	(3)	5	(6)	22	(24)	36
		(01, 05)		(00, 12)		(02, 14)		(16, 34)		(7)
	30,000–40,000	1	(0)	1	(2)	2	(2)	13	(14)	17
		(00, 02)		(00, 09)		(00, 08)		(08, 23)		(3)
	40,000–50,000	1	(0)	0	(0)	1	(1)	1	(1)	3
		(00, 02)		(00, 06)		(00, 07)		(00, 06)		(1)
	> 50,000	3	(1)	0	(0)	2	(2)	9	(10)	14
		(00, 03)		(00, 06)		(00, 08)		(05, 18)		(3)

*Percentage figures, and 95% confidence intervals, shown below the number n and the percentage are per usage category, defined as sessions per day. Data are from 656 riding arenas from a questionnaire in Sweden 2014*.

a *One dollar corresponded to 6.9 SEK in 2014 (https://www.exchangerates.org.uk/USD-SEK-spot-exchange-rates-history-2014.html)*.

### Rider Desired Span for Arena Characteristics

**Figure 4:**
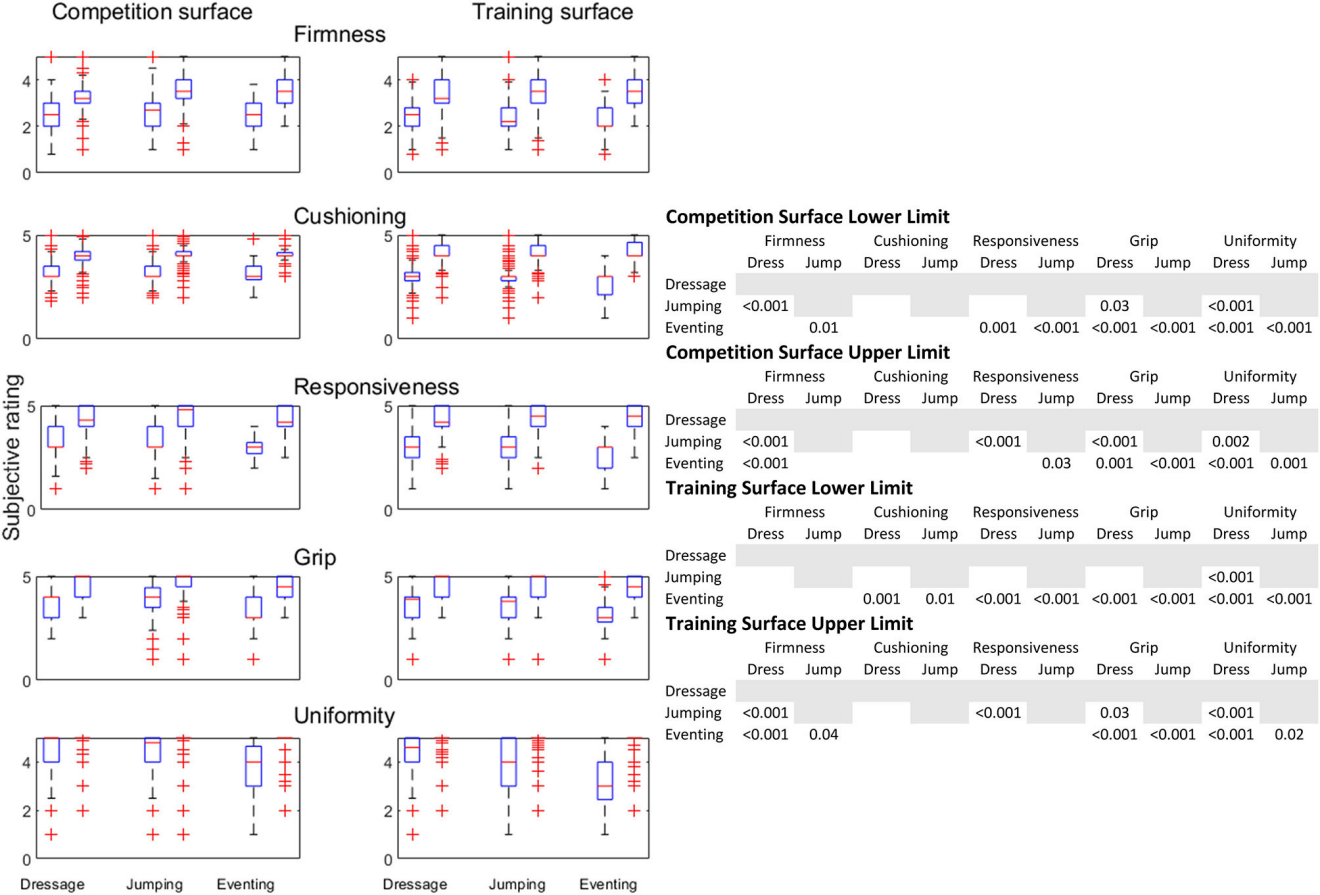
The span of desired property values for competition and training surfaces by primary discipline. Data are from a questionnaire on riding arenas in Sweden 2014. Each pair show the desired low and high property ranges for dressage riders (between 1,024 and 1,417 answers), show-jumping riders (between 988 and 1,449 answers) and eventing riders (between 115 and 147 answers). *P*-values for significant comparisons between the three disciplines are shown to the right. Descriptive statistics in numbers are shown in Supplementary Material 4.

Figure 4 shows the preferred range of arena characteristics from the questionnaires as well as the significance of the comparisons between disciplines. Detailed and descriptive data are provided as Supplementary Material 4. For impact firmness the overall comparisons were significant at group level (*p* < 0.0001) with the exception of the lower acceptable level or lower span on training arenas. The legend in Figure 4 demonstrates, for example, that dressage and show- jumping riders differed significantly in their upper span for impact firmness on both competition and training arenas.

For cushioning only the lower span on training arenas was significant at group level (*p* = 0.003). In the pairwise comparison eventers differed from the other two groups. Eventers stated that a lower limit for the cushioning value was acceptable than other riders.

For responsiveness all four (lower/upper and per competition and training surface) overall comparisons were significant (*p* = 0.002—*p* < 0.0001). Most comparisons with eventers were significant, but the upper spans were shown to be different for dressage and show-jumping riders. The upper rating value for dressage riders was lower than for show-jumping riders.

For grip all four group-wise comparisons were significant (*p* < 0.0001). Show-jumpers wanted the low span at competition arenas to be higher than dressage riders.

For uniformity all four group-level comparisons were significant (*p* < 0.0001) and all pairwise comparisons as well. The upper spans are difficult to decipher from the figure as most respondents stated that a perfect uniformity was optimal. For both competition and training arenas, for the lower limit dressage riders wanted a higher uniformity, show-jumping riders accepted a somewhat lower and event riders the lowest (Figure 4, Supplementary Material 4).

Comparing the lower and upper limits, respectively, for desired spans between competition and training areas, for the characteristics within dressage, show-jumping and eventing all but four of the 30 comparisons were significant (most at *p* < 0.0001). The comparisons that were non-significant were the upper limits for responsiveness in dressage riders and the upper limits in responsiveness, grip and in uniformity for eventers.

## Discussion

### Top-Layer Category and Construction

Both sand-mineral arenas and sand-woodchips arenas occurred in 34% of the data. Sand-mineral arenas were most common outdoors (66%) and sand-woodchips arenas (54%) were most common indoors. After these categories the sand-fibre arenas followed in popularity (14% of all), with fewer arenas found in the group classified as synthetic (8%, Table 1). The arena composition and also the relative allocation likely reflect the situation in Sweden. Establishments deciding on the construction of an arena are likely to opt for designs which are commonly used in other venues with similar usage, weather and climate conditions. It is clear that sand-fibre arenas have gained in popularity as seen in the more recent date of construction (Table 3). Synthetic arenas are generally considered to be synonymous with waxed sand arenas (7). However, in Sweden the waxed arenas actually represent less than half of the synthetic arenas (19 of 50 arenas). The majority of the arenas classified as synthetic in this study had rubber material included in the top-layer.

The distribution of indoor/outdoor arenas will vary with climate. Because of the relatively cold weather in Sweden horses are to a large extent trained in indoor arenas (4). Waxed sands are quite commonly found in the UK (4, 16), where rain is common. The use of wax may be explained by the stated ability to maintain properties over a large range of water content. This may be more important than other characteristics, like consistency over a wider range of temperatures, in areas with more abundant rainfall as in much of the UK. Other reasons for using waxed sand or not, is the availability of sand which is well-suited for mixing with wax as well as “tradition.” In Sweden users may find sand arenas without wax easier to maintain, cheaper, and more sustainable, and more suitable for outdoor arenas which would be subjected to a wide range of temperatures. The absence of wax in many of the arenas also addresses a number of potential problems with disposal of used surfaces. Ready access to wood products in Sweden may also explain the prevalence of wood product mixtures especially in older arenas.

The difficulties encountered with respondents classifying arena top-layer correctly is a clear limitation of the study. Particularly for older arenas, the respondents were less likely to have been directly involved in the construction of the arenas. A number of questions were used which made it possible to better characterise the arenas and the possibility was included for a free text response (not presented in detail here). Notwithstanding the options provided, 62 of the 656 arenas were left unclassified. It is likely that most of these belonged to one of the included categorisations but the information provided was insufficient for classification.

The mean year of construction for the sand-fibre arenas was 2007 which makes these the most recently constructed of all of the categories of top-layers. In contrast the sand-woodchips arenas, had mean year of construction before year 2000 (Table 3). This suggests that new arenas are continuously being built and that some top-layer materials have become increasingly popular. The oldest arena, a sand-woodchips arena, was built in 1945.

Descriptively, grass arenas are wide and cover a large area. Some sand-mineral arenas are relatively large, reflected by that the mean area of this category is larger than the median. Synthetic arenas have a relatively thin top-layer and sand-fibre arenas a slightly thicker second layer. Forces from the hoof landing and weight bearing interacts with deeper layers of the ground. It is therefore important to create uniformity in all layers; top-, mid-, and base-layer. It is interesting that the second layer is thicker with sand-fibre mixtures as top-layer. Since these arenas are more recent it might reflect an increased awareness of the need for a consistent arena surface including the lower layers that provide support for the load associated with both the horses and maintenance machinery.

A rubber layer was found in 54% of the 50 synthetic surfaces, and was also found with other top-layers including sand-fibre and sand-woodchips. In Swedish arenas it is not uncommon to find a layer of rubber underneath the top-layer but above the aggregate base (4). This is thought to provide area elasticity to the surface, by deflection of a larger area of the surface in response to loading (17). A mineral-based composition' Paddex' was found in seven arenas [this is a locally produced mineral-based construct stated to stabilise clay (18)]. A rather small number of the arenas (117/656) was reported to include geotextile. While we asked specifically about the presence of geotextile materials, it is possible that the respondents were unaware of the presence of such materials and that the occurrence was underestimated. For example, sand-woodchips arenas had a low reported frequency of the use of geotextile, but these arenas were also older so memory bias may have created a spurious difference. We also note that geotextile may be used both as separating layer and in pieces mixed into the top layer. The advantage of geotextile as a separating layer is that it stops mixing of different layers. A disadvantage of geotextile as a separating layer is that it may become clogged and prevent drainage. In the case of sand-woodchips arenas the geotextile would have been more likely to the used as a separating layer since woodchips serve some of the same role as geotextile in the top layer. When the geotextile had been used as a separating layer the presence may not have been known to the owner or user.

The more frequent use of a construction company when building a sand-fibre arena, compared to other arenas, is perhaps not surprising (Table 2). The composition of such arenas, including the fibre itself, is often provided by the installing company. Sand-fibre arenas are also newer, and the increased availability of surface expertise also would increase the likelihood that a company would perform the installation. Additionally, since sand-fibre arenas can be more difficult to instal and usually represent a more expensive top-layer, specific expertise for installation would be more likely to have been employed. A number of arenas also had the ground prepared during construction (Table 2). Obvious reasons not to prepare the ground include that the construction area is already flat, the arena may be a natural grass arena or the ground preparation may have been performed previously for another purpose. The respondents reported that 30% of the arenas had drainage present (Table 2). Reasons not to report drainage include location of the arena on high ground, an indoor arena, or not being aware of drainage laid during construction. From only a small number of arenas it was reported that sieve curves were used to select the sand during construction. In this case it can be speculated that such information was used on more surfaces, but that this decision making process was not clear to the respondent. Most arenas, irrespective of top-layer composition, or usage frequency, had a person with assigned maintenance responsibility.

### Usage of Arenas

For indoor arenas, the number of daily sessions was not influenced by type of arena (Figure 1). For outdoor arenas, a substantial share of outdoor sand-mineral arenas (138 of 161) were only used for 1–10 sessions per day, likely often representing arenas used as a secondary arena. However, the respondents were selected as high-level competitors or from large establishments, and our sample did not aim to directly represent the private sand-minerals arenas found in Sweden. A mistake was made in the coding of the daily number of sessions for the second arena, thus only four usage categories could be used for the analysis (Supplementary Material 2).

As expected, the most common usage was dressage and show-jumping, although all 11 listed disciplines were represented (Table 8). The most common arenas used in competition were indoor sand-woodchips followed by indoor sand-fibre arenas. In relation to their relatively high occurrence, many outdoor sand-mineral arenas (102 of 161) were never used for competition. This corresponds with their low number of sessions (Figure 2) and with the suggestion that they may often be used as secondary arenas.

Despite show-jumping being the largest competition discipline (19) the most common primary arena usage was dressage (Table 8). This could reflect that also show-jumpers often do their main part of training as flat work/dressage. When indoor arenas are used for show-jumping competition, such as at high speeds and sharp turns, they would require a surface that is more stable and has more grip. This type of surface will require less maintenance during a competition and allow faster riding without risk of the hoof sliding on the surface. This is not necessarily beneficial to the locomotor apparatus, since increased speed and grip will increase forces between the ground and the hoof (20).

If using the numbers from Figure 1 (taking the median number in each session category and multiplying with the counts [yielding e.g., >3,200 sessions in indoor sand-woodchips, >1,400 in outdoor sand arenas and >1,900 in indoor sand-fibre arenas]), this may approximate the load of sessions on the studied arenas. From such calculations, most sessions were performed on sand-woodchips arenas, followed by outdoor sand-mineral arenas and indoor sand-fibre arenas at about half of the level for the sand-woodchips arena. This very crude calculation thus suggest for example that many horses are daily exposed to older arena categories. As 65% of the surface responses (432/656) came from competition riders, this, as well as the numbers in Figure 2 suggest that many competitions are still held on sand-woodchips arenas.

### Maintenance

Arenas were commonly maintained by dragging or harrowing, but less frequently by deep harrowing (Table 4). Excluding grass surfaces, dragging, or harrowing were used in the majority of the specific arena top-layer categories at least once a week. Deep harrowing was reported as being performed once a week; in 12, 18, and 13% of the sand-mineral, sand-woodchips, and sand-fibre arenas, respectively, and more frequently in some arenas. That deep harrowing was so frequently done in sand-fibre arenas was unexpected, as sand-fibre arenas are generally considered as needing less maintenance. While suppliers of market sand-fibre arenas claim these as needing less maintenance, it may reflect that these arenas are used more often or that the owner wants to keep the arenas in top condition. It is likely that this more frequent maintenance, e.g. deep harrowing, will provide a more uniform arena and more controllable functional properties (11).

Rolling was used relatively infrequently. Rolling is typically recommended for sand-fibre arenas but also for most other arena types (11). Commercial arena maintenance machinery for sand-fibre mixtures often include three different mechanisms on a single maintenance machine. The sections of the maintenance machine include a drag that evens out the surface, a harrow that opens and mixes the top-layer and a roller that repacks the material. The reason for low rolling usage frequency found in this survey may have been either a poor understanding of the terminology or limited awareness of the importance of the technique. However, it may also indicate that the function of the combination machine is not well-understood by the respondents.

Manure was removed from 90% of all arenas. Prudent manure removal may lead to increased sustainability of arenas. Manure removal was contrasted to usage frequency and there were no large differences among the top-layer categories (on grass arenas somewhat fewer (69%) removed manure) or usage groups.

### Watering and Watering Technique

While most arenas were watered the methods varied. In 23% of the arenas a sprinkler system was used, 22% were watered using a water tank and 58% were watered by hose (Table 5). Sand-fibre arenas had sprinkler systems more often than sand-mineral and sand-woodchips arenas, perhaps because they were newer designs. Arena water content obviously also depend on whether arenas are irrigated. A large share of all represented top-layers were salted, except for the grass arenas. Sand-woodchip arenas were salted more often (68%) than sand-mineral arenas (37%). Salting of arenas will be done in winter-time to prevent freezing, mainly of indoor arenas, and in summer time to decrease the dust content of the air, mainly of outdoor arenas (11). Sand-woodchips arenas may have been relatively more common in establishments in more northern locations with a colder climate needing salting at winter time. Sand-woodchips arenas are also likely to create a dustier environment than other top-layers, suggesting that they could benefit from salting even in the summer. Sand-woodchips arenas may also be more commonly used as riding school arenas with greater budget constraints. That the sand-woodchips arenas were relatively older (Table 3) is consistent with both the budget constraints for these type of establishments and that they are often found more frequently in locations where winters are harsher (North and inland within the country).

Without considering rainfall, outdoor sand-fibre arenas were watered more during the summer and spring-autumn, than other outdoor top-layers. Indoor sand-fibre arenas were also watered more during the winter than other top-layers. Sand-fibre top-layers, as most top-layers, need water to be controlled in order to maintain appropriate surface characteristics. The sand used in sand-fibre arenas designs allows water to pass easily though the surface. This type of free draining surface may require greater water addition to maintain moisture content for the desired functional properties. With increasing number of sessions per week watering was also done more often (Table 6). Depending on construction (particle size distribution in the top-layer etc.) water holding capacity can vary a lot. However, several days without watering, or rainfall, will clearly lower moisture content in the top-layer. Several functional properties are affected by moisture content (21). It is therefore likely that surface properties will vary considerably over time in many riding arenas. Subjecting horses to variation in functional properties of the ground that horses are worked on can be theoretically beneficial. Variation in training, including variation in surface properties, may prepare the horse for changes in surfaces which would be encountered at a competition or other performance (4). However, if the objective is to keep the arena consistent over time, a large number of arenas may not be watered at sufficient frequency.

The mechanical behaviour of an arena surface is not only affected by material composition, but also highly affected by the maintenance such as watering and harrowing (22, 23). The same maintenance measure has been shown to produce different effects on different arena materials (16). The most cost effective way to improve surface properties is likely often to increase frequency of and the quality of maintenance. But, resources (economical and/or personnel) are often scarce at horse yards and the know-how might be limited in terms of how maintenance should be performed or how important it is. Limited epidemiological information exists that describes how arena maintenance is performed on different yards (24).

### Economics and Arena Sustainability

Questions were included about the frequency of top-layer renewal, the expected lifetime of the arenas and the expected annual cost of maintaining arenas. These questions have been analysed both with respect to the top-layer composition and the category of usage (Tables 7, 9). Summarising, the majority of arenas are expected to require frequent renewal, either every second to fourth year (39%) or every fifth to tenth year (29%). For few high-usage arenas (>40 sessions per day) it was responded that the top-layer would require infrequent renovation (2%) and many stated that renovation is expected on an annual basis (42%). The anticipated frequency of top-layer renovation is relatively consistent among the top-layer categories. One exception is sand-woodchips arenas that are renewed more frequently than at 10 year intervals.

In the highest usage category (Table 9) respondents expected the duration of use for the arenas to be 1–3 years for 16% (of the high usage arenas), 4–6 years for 41%, 7–9 years for 12%, 10–12 years for 24%, 13–15 years for 2%, and over 15 years for 5%. Comparing expected costs among the highest usage categories (Table 9), yearly expected costs for the arenas were 0-1,000 SEK 2%, 1,000–5,000 SEK 16%, 5,000–10,000 SEK 14%, 10,000–20,000 SEK 18%, 20,000–30,000 SEK 24%, 30,000–40,000 SEK 14%, 40,000–50,000 SEK 1%, and >50,000 SEK 10%. Hence few of the highest usage arenas were described as being expected to have a long expected lifetime, but few expected a “high” yearly cost. Our perception is, that in general, and across usage categories, the expected costs may be too low to optimally maintain the arenas for the intended activities. However, the questions may be difficult to answer correctly unless it is the person with direct financial responsibility that addresses the question. Since many of the respondents may not be financially responsible, the actual costs are likely to be higher. It is beneficial if costs are transparent to both the people responsible for arena maintenance and the riders. The investment in surfaces is important both for management of horse health and for creating a sustainable environment. These answers may also suggest the potential benefit of increased education and awareness of how arenas change over time. For example the sieve curve of sand particles will be shifted toward finer material with greater usage of the arena with a resulting degradation in surface properties (11). Thus, an increase in the amount of fine material will increase the compaction and reduce the drainage capacity. Top-layers will become worn and unevenly distributed with the result that the top-layer will require addition of material and redistribution of material over the base. Sand-woodchips surfaces were rated as most in need of renewal. However, the sand-woodchips arenas were also somewhat older so this may be simply a reflection of circumstances. In many instances regular renewal had been undertaken at many of these arenas and it was actual experience and not more-general guidelines that guided answers. It may also reflect that this is an ongoing issue with these types of surfaces. Woodchips, including chips and bark, degrade relatively quickly, on the other hand woodchips are relatively inexpensive in Sweden. Thus, even if frequent renewal is required the overall cost may be reasonable. If dragging, harrowing, deep harrowing, and rolling are counted together (Table 6) it is found that the majority of arenas are maintained at least once a week. Disregarding arena composition, arenas used more infrequently are maintained less often (Table 9). Further research into the field of longevity of equestrian arenas and the durability of the layers relative to usage and maintenance, is needed. Growing interest for sustainability in sport surfaces is evidenced by, for example, international organisations targeting “environmentally sympathetic” renewal and disposal of synthetic turf sport surfaces (25).

### Riders and Rider Preferences

Riders rated the desired surface properties (Supplementary Material 1) using descriptions based on development and research of surface properties (6, 7). The main disciplines for these riders were dressage and show-jumping, followed by eventing. Only a small number of respondents were from other disciplines. The specific comparisons of desired properties were limited to the three most highly represented disciplines.

Differences between dressage and show-jumping riders include that show-jumping riders accepted a higher upper span for impact firmness on both competition and training arenas, compared to dressage riders (Figure 4). Dressage riders rated the upper span for responsiveness lower compared to ratings from show-jumping riders, both for competition and training. For grip show-jumpers wanted the low span at competition arenas to be higher than what was rated by the dressage riders. For the lower span of uniformity dressage riders wanted a higher uniformity while show-jumping riders accepted a somewhat lower span for both competition and training arenas. Including eventing in the comparison, at competition eventers stated a lower span cushioning value than other riders and eventers stated the lowest values for the low span of uniformity.

Many of the results found were consistent with the expectations. The show-jumping rider cantering through a tight turn at a relatively high speed needs a footing with enough grip and firmness to avoid slipping. This, and that many sand-fibre arenas are recently constructed, may suggest that the show-jumping rider often prefers a sand-fibre arena for competition and at least for part of the training. This is somewhat in contrast to that competitions were often held on sand-woodchips arenas in 2014 (Figure 2). A reasonably high responsiveness will also retain energy from canter strides and jumps. The rider's expectations for the performance of the surface will be a mixture of peer influence, what research suggest and to a large extent the rider's own experience. It is likely that the rider's experience with different types of footings through their career will lead riders from different disciplines to focus on different aspects of the performance. However, the optimal footing may, at least at times, be truly different for different disciplines. The differences between disciplines is an area that is not easy to systematically investigate. A fact is that the rider will have to evaluate the properties through the much-heavier horse and not by a walking human, as the visco-elastic properties of interest are load-dependent (20). Previous work suggests that riders may have difficulties discerning between properties or terminology, in particular separating firmness and cushioning may be difficult (6).

When comparing between training and competition arenas, most comparisons within dressage and show-jumping were significant. The exception is the upper span of responsiveness in dressage riders, indicating that high responsiveness is desired in both training and competition. This suggests that riders have experience or opinions that competition and training arenas could, or should, without detrimental effects on the horses or consequences for the competition results, differ in their properties. Our expectations included a more narrow range of surface properties for competition surfaces compared with training arenas. The lower span for cushioning at competition appears to be more narrow (Supplementary Material 4), both for dressage and show-jumping riders, but such findings (the shape of the distributions) have not been formally, i.e., statistically, evaluated.

A more evidence-based understanding will require more training in the equestrian community to understand functional properties, as well as to improve the subjective evaluation of surfaces, while riding (6). This is necessary when planning how to train on different footings, but also for the development of specifications for new arenas. The planning, conducting and evaluation of the arena maintenance will also affect not only the performance but the environmental impact of the arena surface material.

The study did unfortunately not address how riders rated welfare of the horses when riding on the surfaces vs. how they perceived the economy and sustainability of the arenas (i.e., the two datasets were not linked for any analyses). However, when a show-jumping rider wants a relatively high grip, reasons include safety for horse and rider when riding tall fences, but also the possibility to overcome such obstacles and do so quickly. At higher levels, horses, equipment and training are all expensive. This is in contrast to at the base-level, e.g., at a riding school the sport is still relatively expensive to perform, but the costs are considerably lower. At such base-level the horses are not subjected to the tallest fences or to demanding dressage exercises such as canter pirouettes; but they are instead likely performing low-intensity work during longer periods of time, which is likely also a challenge for the locomotor apparatus. However, the consideration of topping-up an older sand-woodchip surface or building a new sand-fibre arena may be different for these groups. Of course, there are also establishments that have both a riding school and horses boarded “privately owned,” that may strive to achieve high-level competition results.

### Comments on the Design of the Study

Given the complicated questions posed the response rate was reasonably good, 21 and 15% for the two registries, these registries having assumed adequate and complete coverage, respectively. However, free text answers reflected that many respondents felt that increasing knowledge and awareness of surface questions is important to horse health and performance (data not shown).

Some issues should be considered when evaluating the study. It is possible that more than one response was received for some of the arenas, given that questionnaires were distributed both to high-level riders and directly to the management of the riding establishments. While the possibility exists for multiple responses, it is unlikely that duplicate answers were provided to any large degree as questionnaires were addressed to those responsible for the arenas. Given the recent focus on arena properties and the large number of new arenas having been built in Sweden during the beginning of the 21st century (unpublished information), there is also a possibility that we achieved different response rates among top-layer categories. However, we consider it likely that there is a significant focus on arena properties in large establishments irrespective of the age of the arenas and other factors. The questions were relatively difficult and there were many missing answers, at the same time many times verification of information was possible through several questions. For example, asking whether geotextile was found between the 1st and 2nd layer, as well as between the 2nd and 3rd, we think we captured more correct data regarding the presence of geotextile and the same time appreciating the resolution of the awareness of the persons responsible for the arenas. The number of missing values also indicates that even the persons responsible for the arenas may not have specific knowledge of the arena construction, especially for construction details that occurred some time ago. For example, in 42% (Table 2) of all arenas, respondents could not state if sieve curves were used when constructing the arena. It is also possible that some respondents have confused some of the response categories such as harrowing and deep harrowing. The result would most likely have been that the true differences in frequency between these two categories have become understated. We note that, as the targeted respondents were the persons responsible for the arenas at each establishment, e.g., the terms dragging (in Swedish “sladdning”), harrowing (“ytlig harvning”), and deep harrowing (“djup harvning”) were not further explained (Supplementary Material 2).

Another methodological issue concerns the statistics. To be able to present the material, statistical tests have been performed for selected questions. Most statistics are based on the non-overlap of 95% confidence interval and for a few questions no statistical methods were used. Multivariable methods have not been used, neither any corrections for multiple comparisons.

### Future Research

Sustainability is important in two very different aspects when discussing arenas. One is from the perspective horse health and the other is the environmental impact of sand and the use of footing material additives such as wax. It would have been optimal to gather information on horse health through this questionnaire. However, our previous experience gathering information on for example horse health in riding schools (26, 27), or from horses at top-level show-jumping riders (4), suggested to us that such information would a priori need commitment from each horse owner [not just the person in charge of the arena] and considerable efforts of standardisation to compare among respondents. Still, the equestrian community needs arenas that protect the locomotor apparatus of the horses, both at training and at competition. Unfortunately, scant data exist on how equestrian arenas should be constructed or maintained to have generally positive health outcomes and whether various surfaces can optimise horse health for the general horse. Murray et al. (3) found, using a large population of British dressage riders, that using a sand-surface was associated with more lameness, but that this effect lessened when the horse was worked more frequently on the sand surface. In order to study the locomotor injury consequences of surface usage; training amount, intensity and surface usage must be monitored, at the same time that surface properties are measured. To develop the required data for a large number of horses over time ridden on various surfaces is a huge undertaking. For extrapolation of such results, it is also important that data are gathered from multiple disciplines, including general riding horses, and from several continents. A limited number of studies on arenas, in some way including the properties; experimental (e.g., 6), cross-sectional (3), or longitudinal (4), have been undertaken, and such studies will also enhance interpretation. Furthermore, sand use must be sustainable since natural sand is a limited resource. Additional environmental impacts must also be considered such as sand transports, the disposal of materials during renovation and potential loss of materials into the environment during use. In the current study few respondents (*n* = 33) were aware of a plan for disposal of used arena material. How to build, maintain and dispose of sport surfaces; which equestrian sports surfaces to some degree have in common with other sport surfaces, also needs to be documented and associated research performed (7).

### Conclusion

Data on 656 equestrian surfaces in Sweden in use in 2014 have been presented. Sand-mineral arenas were most common outdoors and sand-woodchips arenas most common indoors, followed by sand-fibre arenas and even fewer synthetic arenas. Most likely a large number of arena owners largely under estimate the resources necessary to properly maintain the consistency of the arena over time. Show-jumping and dressage riders perceive ideal surface characteristics somewhat differently. For example show-jumping riders preferred higher impact firmness. Improved knowledge of maintenance methods, timing, and priorities for arenas are important both to the users and arenas managers. It is important that construction companies or arena managers commit more to horse-oriented research of equestrian surfaces, ongoing education of riders must also be undertaken. Future consideration of new arenas and research into organic arena management is important in order to minimise the impact of equestrian arena surfaces on the environment and to optimise the health of the horse.

## Data Availability Statement

The original contributions presented in the study are included in the article/Supplementary Material, further inquiries can be directed to the corresponding author/s.

## Ethics Statement

Ethical review and approval was not required for the study on human participants in accordance with the local legislation and institutional requirements. Written informed consent for participation was not required for this study in accordance with the national legislation and the institutional requirements.

## Author Contributions

This study was initiated by LR, ML, and EH. It was designed by LR, EH, and AE and analysed by AE. Drafting and compiling was done by AE, supported by LR, ML, EH, and MP. The final manuscript was approved by all authors.

## Funding

This work was supported by SvFR.

## Conflict of Interest

The authors declare that the research was conducted in the absence of any commercial or financial relationships that could be construed as a potential conflict of interest.

## Publisher's Note

All claims expressed in this article are solely those of the authors and do not necessarily represent those of their affiliated organizations, or those of the publisher, the editors and the reviewers. Any product that may be evaluated in this article, or claim that may be made by its manufacturer, is not guaranteed or endorsed by the publisher.

## References

[1] Hernandez J Hawkins DL Scollay MC. Race-start characteristics and risk of catastrophic musculoskeletal injury in Thoroughbred racehorses. J Am Vet Med Assoc. (2001) 218:83–86. 10.2460/javma.2001.218.83 11149721

[2] Parkin TDH Clegg PD French NP Proudman CJ Riggs CM Singer ER. Race- and course-level risk factors for fatal distal limb fracture in racing Thoroughbreds. Equine Vet J. (2004) 36:521–6. 10.2746/0425164044877332 15460077

[3] Murray RC Walters JM Snart H Dyson SJ Parkin TDH. Identification of risk factors for lameness in dressage horses. Vet J. (2010) 184:27–36. 10.1016/j.tvjl.2009.03.020 19369100

[4] Egenvall A Tranquille CA Lönnell AC Bitschnau C Oomen A Hernlund E. Days-lost to training and competition in relation to workload in 263 elite show-jumping horses in four European countries. Prev Vet Med. (2013) 112:387–400. 10.1016/j.prevetmed.2013.09.013 24125697

[5] Peterson ML Reiser RF Kuo PH Radford DW McIlwraith CW. Effect of temperature on race times on a synthetic surface. Equine Vet J. (2010) 42:351–7. 10.1111/j.2042-3306.2010.00072.x 20525055

[6] Hernlund E Egenvall A Hobbs SJ Peterson ML Northrop AJ Bergh A. Comparing subjective and objective evaluation of show jumping competition and warm-up arena surfaces. Vet J. (2017) 227:49–57. 10.1016/j.tvjl.2017.09.001 29031331

[7] Hobbs SJ Northrop AJ Mahaffey CA Martin JH Clayton HM Murray RC. Equine Surfaces White Paper. FEI books (2014). Available online at: http://www.fei.org/system/files/EquineSurfacesWhitePaper.pdf (accessed July 19, 2021).

[8] Peterson ML McIlwraith CW Reiser RF. Development of a system for the in-situ characterisation of thoroughbred horse racing track surfaces. Biosyst Eng. (2008) 101:260–9. 10.1016/j.biosystemseng.2008.07.007

[9] Northrop AJ Hobbs SJ Holt D Clayton-Smith E Martin JH. Spatial variation of the physical and biomechanical properties within an equestrian arena surface. Proc Eng. (2016) 147:866–71. 10.1016/j.proeng.2016.06.288

[10] Hitchens PL Morrice-West AV Stevenson MA Whitton RC. Meta-analysis of risk factors for racehorse catastrophic musculoskeletal injury in flat racing. Vet J. (2019) 245:29–40. 10.1016/j.tvjl.2018.11.014 30819423

[11] Hernlund E Lönnell C Roepstorff L Lundholm M Bergström L Andersson M. Equestrian Surfaces – A Guide. Svenska Ridsportförbundet (2014). Available online at: http://pub.epsilon.slu.se/id/eprint/12229 (accessed November 15, 2021).

[12] Bridge JW Mahaffey C Peterson ML. Analytical test methods used to characterize granular composite sport surface materials. Appl Mech Mater. (2014) 440:74–81. 10.4028/www.scientific.net/AMM.440.74

[13] Bartlett R. Sports Biomechanics: Preventing Injury and Improving Performance. 2nd ed. New York, NY: Routledge (1999).

[14] van Weeren PR. On surfaces and soreness. Vet J. (2010) 186:129–30. 10.1016/j.tvjl.2010.09.006 20933447

[15] Netigate. Netigate (2021). Available online at: http://www.netigate.se (accessed August 20, 2021)

[16] Tranquille CA Walker VA Hernlund E Egenvall A Roepstorff L Peterson ML. Effect of superficial harrowing on surface properties of sand with rubber and waxed-sand with fibre riding arena surfaces: a preliminary study. Vet J. (2015) 203:59–64. 10.1016/j.tvjl.2014.10.027 25510315

[17] Nigg BM Yeadon MR. Biomechanical aspects of playing surfaces. J Sports Sci. (1987) 5:117–45. 10.1080/02640418708729771 3326948

[18] SSAB. Paddex (2021). Available online at: http://www.paddex.se (accessed August 20, 2021).

[19] SvRF. (2014). Available online at: https://www.ridsport.se/globalassets/svenska-ridsportforbundet/dokument/forbund/verksamhetsberattelser/ridsport_siffror_2014.pdf (accessed November 15, 2021).

[20] Thomason JJ Peterson ML. Biomechanical and mechanical investigations of the hoof-track interface in racing horses. Vet Clin North Am Equine Practice. (2008) 24:53–77. 10.1016/j.cveq.2007.11.007 18314036

[21] Holt D Northrop A Owen A Martin J Hobbs SJ. Use of surface testing devices to identify potential risk factors for synthetic equestrian surfaces. Proc Eng. (2014) 72:949–54. 10.1016/j.proeng.2014.06.160

[22] Peterson ML McIlwraith CW. Effect of track maintenance on mechanical properties of a dirt racetrack: a preliminary study. Equine Vet J. (2008) 40:602–5. 10.2746/042516408X330347 19031517

[23] Setterbo JJ Yamaguchi A Hubbard M Upadhyaya SK Stover SM. Effects of equine racetrack surface type, depth, boundary area, and harrowing on dynamic surface properties measured using a track-testing device in a laboratory setting. Sports Eng. (2011) 14:119–37. 10.1007/s12283-011-0073-4

[24] Murray RC Walters J Snart H Dyson SJ Parkin TDH. How do features of dressage arenas influence training surface properties which are potentially associated with lameness? Vet J. (2010) 186:172–9. 10.1016/j.tvjl.2010.04.026 20888276

[25] ESTC ESTC Guide Processing End of Life Synthetic Turf Sports Surfaces 2021, Edition,. (2021). Available online at: https://www.estc.info/knowledge-centre/processing-end-of-life-synthetic-turf-sports-surfaces/ (accessed November 19, 2021)

[26] Egenvall A Lönnell C Roepstorff L. Analysis of morbidity and mortality data in riding school horses, with special regard to locomotor problems. Prev Vet Med. (2009) 88:193–204. 10.1016/j.prevetmed.2008.10.004 19042047

[27] Egenvall A Lönnell C Johnston C Roepstorff L. Orthopaedic health status of horses from 8 riding schools - a pilot study. Acta Vet Scand. (2010) 52:50. 10.1186/1751-0147-52-50 20727185 PMC2939618

