# Supplementary material for: The Descriptions and Attitudes of Riders and Arena Owners to 656 Equestrian Sport Surfaces in Sweden
Source: Front Vet Sci. 2021 Dec 23;8:798910. doi: 10.3389/fvets.2021.798910 (PMC8732755; doi:10.3389/fvets.2021.798910)
Supplement: Supplementary file 1 [file Data_Sheet_1.PDF]

Supplementary Data 1:

Table S1. Questionnaire to the riders on perceived optimal surface properties. Translated (in some instances taking differences in cultural usage of terms into account) from Swedish.

Which is your main discipline?

Dressage

Show-jumping

Other

If other discipline, please indicate which:

CRITERIUM 1: Firmness

How firm/loose is the top layer? How much can the hoof rotate in the surface?

With regards to the characteristics of the top surface please provide a description on the following scale that you think is describes the competition and training surfaces, respectively.

1 = The surface is very loose. The hoof rotates easily in the surface. The hoof leaves a depression in the surface with minimal contours. E.g. the top layer is similar to dry beach sand.
2 = The hoof leaves an impressions where it is possible to detect the vague shape of a hoof. E.g. like slightly wet turf.
3 = The hoof leaves a well defined imprint in the surface. The sole and the frog can be distinguished in the imprint. (When the horse pushes off a "heel" is built up in the surface behind the impression of the hoof.)
4 = The hoof leaves an imprint mainly from the shoe. You can clearly hear the sound of the hoof when it impacts the surface.
5 = The surface is very firm. No imprints or only the rim of the shoe is evident. E.g. like a firm gravel or dirt road or tarmac road.

EXAMPLE: If you think a good competition surface is around a 3, your answer may look like this (in Swedish):

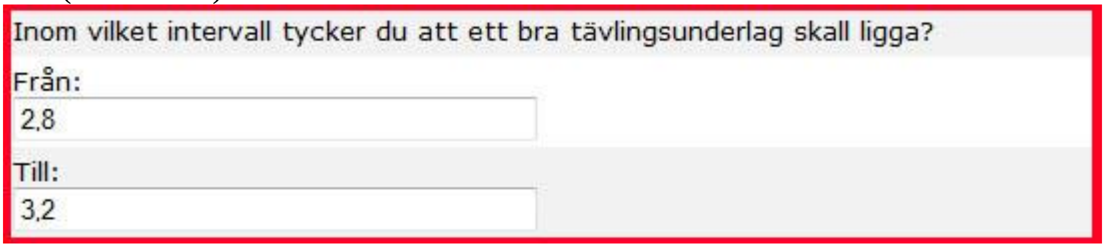
Inom vilket intervall tycker du att ett bra tävlingsunderlag skall ligga?

Från:

2,8

Till:

3,2

Within what interval do you think an optimal competition surface should be rated?

From:

To:

Within what interval do you think an optimal training surface should be rated?

From:

To:

CRITERIUM 2: Cushioning (in Swedish dämpning)

How much of the maximal loading is cushioned? This describes the response of the surface after the mass of the horse is transferred to the hoof. The ***amount*** *of cushioning is evaluated as described below. (The type of cushioning is described in the following question.)*

1 = The footing is very stiff. E.g. after the initial loading of the surface the arena is like concrete.
2 = The footing is moderately stiff. E.g. similar to a turf track with a high percentage of clay that has dried.
3 = The footing has a limited degree of cushioning. E.g. Sand based arena with a certain amount of "giving in" of the top layer but without any deep compliance.
4 = A footing that has a moderate/ obvious cushioning. The cushioning of the maximal loading (at least) partly derives from pliancy in deeper layers. E.g. this could either be an optimal turf track or an artificial (geo textile / wax-coated) surface with a deeper loose layer of sand or sand mixed with rubber. It could also be a relatively deep sand based arena.
5 = The ground is very cushioned (deep/loose or elastic). E.g. either like a deep, dry sand dune or like an extremely elastic footing, e.g. rubber material with very good elasticity in deeper layers.

Within what range do you think an optimal competition surface should be rated?

From:

To:

Within what range do you think an optimal training surface should be rated?

From:

To:

CRITERIUM 3: Responsiveness (in Swedish Elasticitet)

How much of the cushioning is achieved by elasticity of the ground?

In the previous question you described the amount of cushioning in the footing. Now you are asked to describe how much of the cushioning that returns energy to the horse i.e. the elastic response.

1 = Totally non-elastic cushioning. No energy is given back to the horse. (Dead ground.)
E.g. very loose and deep sand, like dry washed sand with no binding material.
2 = About 25% of the cushioning is elastic.
3 = Approximately 50% of the cushioning is elastic
4 = Mainly, ~75% elastic cushioning.
5 = Very elastic most of the energy is returned to the horse (a "tuned" elasticity). E.g. arena with fibre-sand that is very elastic in the top layer. Another example is arenas constructed with rubber layer deeper in the construction.

Within what range do you think a good competition surface should be rated?

From:

To:

Within what range do you think a good training surface should be rated?

From:

To:

CRITERIUM 4: Grip

How good is the grip?

1 = The footing is very slippery. You would not ride on it without studs.
2 = The horse would slip if you turn in high-speed or make relatively sharp turns. It will also slip at push off now and then. (You would probably not choose to make the horse jump on the footing without studs.)
3 = The hoof slides slightly in the landing. The hoof doesn't slip at push-off during trotting and cantering in moderate speeds and turns. (The footing permits jumping but you would maybe not do a jump off on it without studs.)
4 = The horse rarely slips at hard breaking, sharp turns or at push-off.
5 = Extremely good grip, the horse "never" slips whatever the way you ride.

Within what range do you think an optimal competition surface should be rated?

From:

To:

Within what range do you think an optimal training surface should be rated?

From:

To:

CRITERIUM 5: Uniformity

How uniform/consistent is the footing, including all previous properties over the entire arena? Grade from one to five where:

1 = Large variation between different parts of the arena, i.e. very deep or slippery in certain parts of the arena.
2 -4 = In between 1 and 5
5 = Very uniform, all properties are the same all over the arena.

Within what range do you think an optimal competition surface should be rated?

From:

To:

Within what range do you think an optimal training surface should be rated?

From:

To:

Any more thoughts or opinions?:
