# Supplementary material for: The Descriptions and Attitudes of Riders and Arena Owners to 656 Equestrian Sport Surfaces in Sweden
Source: Front Vet Sci. 2021 Dec 23;8:798910. doi: 10.3389/fvets.2021.798910 (PMC8732755; doi:10.3389/fvets.2021.798910)
Supplement: Supplementary file 2 [file Data_Sheet_2.PDF]

Supplementary Data 2:

Table S2. The arena questionnaire (translated from Swedish)

Arena 1

In this section, we ask you to answer questions related to the main arena (arena 1). In the following section, we will ask you to answer questions that related to arena 2. The second section will only apply to facilities with multiple arenas and should be omitted if they are not applicable.

BACKGROUND AND USAGE

Description/name on the arena (e.g. indoor arena, grass arena)

○ Indoor arena
○ Outdoor arena

Year arena was built

Length in meters

Width in meters

What activities are performed at the establishment?

	Yes	No
Riding school	○	○
Competition	○	○

Training	○	○
Education/training of horses	○	○
Horse boarding	○	○
Other	○	○

Average number of daily sessions on the arena (one horse during 45-60 min is defined as one session)- OBS- for the second arena the alternatives were instead 1-5, 6-10, 11-20, 21-30, 31-40, 41-50 and > 50, this was a mistake and limited the number of categories that could be used in the presentation.

○	1 - 10
○	11 - 20
○	21 - 40
○	41 - 60
○	62 - 80
○	81 or more

What disciplines are represented on the establishment? (rank, 1 for the most common)

	1	2	3	4	5	6	7	8	9	10	11	12
Show-jumping	○	○	○	○	○	○	○	○	○	○	○	○
Dressage	○	○	○	○	○	○	○	○	○	○	○	○
Eventing	○	○	○	○	○	○	○	○	○	○	○	○
Driving	○	○	○	○	○	○	○	○	○	○	○	○
Voltige	○	○	○	○	○	○	○	○	○	○	○	○
Working Equitation	○	○	○	○	○	○	○	○	○	○	○	○
Gymkhana	○	○	○	○	○	○	○	○	○	○	○	○
Western	○	○	○	○	○	○	○	○	○	○	○	○
Reining	○	○	○	○	○	○	○	○	○	○	○	○
Icelandic horses	○	○	○	○	○	○	○	○	○	○	○	○
Endurance	○	○	○	○	○	○	○	○	○	○	○	○

If competitions are arranged on the establishment – what is the highest level for each discipline?

	Local	Regional	National	Elite	International
Show-jumping	○	○	○	○	○
Dressage	○	○	○	○	○
Eventing	○	○	○	○	○
Driving	○	○	○	○	○
Endurance	○	○	○	○	○
Voltige	○	○	○	○	○
Working Equitation	○	○	○	○	○
Gymkhana	○	○	○	○	○
Reining	○	○	○	○	○
Western	○	○	○	○	○
Icelandic horse competition	○	○	○	○	○

How often are competitions held on the surface?

- ○ Never
- ○ <4 times /year
- ○ 5-9times/year
- ○ 9-20 times/year
- ○ >20 times/year

Is the arena used for warm-up at competitions?

- ○ Never
- ○ Sometimes
- ○ Often
- ○ Always

CONSTRUCTION

Was a company hired for the arena construction?

- ○ Yes
- ○ No
- ○ Don’t know

How was the ground prepared during the original construction?

- ○ Excavation and replacement of native soil
- ○ Drainage added to bottom layer
- ○ Don’t know

☐

	Sand (from natural sources)	Crushed sand material (manufactured sand)	Fibre	Rubber	Waxed sand	Wood chips	Other
Top-layer content:	☐	☐	☐	☐	☐	☐	☐

Top-layer thickness in cm:

[ ]

The most common diameter of the sand particles in the top-layer. Dimensions are obtained from particle size analysis typically using sieves.

[ ]

☐

	Sand-mineral (from nature)	Stone dust	Bearing layer of crushed material	Rubber	Geotextile	Drainage hose/tube	Other
Layer 2 content:	☐	☐	☐	☐	☐	☐	☐

Layer 2, thickness in cm:

The dimension of the stone material in layer 2. Dimension implies the particle size from a sieve analysis.

	Sand-mineral (from nature)	Stone dust	Bearing layer of crushed material	Rubber	Geotextile	Drainage hose/tube	Other
Layer 3 content:	☐	☐	☐	☐	☐	☐	☐

Layer 3, thickness in cm:

The dimension of the stone material in layer 3. Dimension implies the particle size from a sieve analysis.

	Sand-mineral (from nature)	Stone dust	Bearing layer of crushed material	Rubber	Geotextile	Drainage hose/tube	Other
Layer 4 content:	☐	☐	☐	☐	☐	☐	☐

Layer 4, thickness in cm :

The dimension of the stone material in layer 4. Dimension implies the particle size from a sieve analysis.

	Sand-mineral (from nature)	Stone dust	Bearing layer of crushed material	Rubber	Geotextile	Drainage hose/tube	Other
Layer 5 content:	☐	☐	☐	☐	☐	☐	☐

Layer 5, thickness in cm:

The dimension of the stone material in layer 5. Dimension implies the particle size from a sieve analysis.

Were sieve curves or other specifications used when ordering the material?

- Yes
- No
- Don’t know

MAINTENANCE

Is there a person responsible for the arena surface (ie a person that decides what and when actions are taken)?

- Yes
- No

Continuous maintenance:

What type of maintenance is undertaken on the arena and how often is it done?

	Each day	4-6 times a week	2-3 times a week	once a week	More seldom
Dragging	○	○	○	○	○
Harrowing	○	○	○	○	○
Deep harrowing	○	○	○	○	○
Rolling	○	○	○	○	○
Watering	○	○	○	○	○

Other ○ ○ ○ ○ ○

Is there a sprinkler system for watering?

○ Yes

○ No

Is the surface watered by tank?

○ Yes

○ No

Is the surface watered manually

○ Yes

○ No

How often is the surface watered per month during spring/autumn?

[ ]

How often is the surface watered per month during summer??

[ ]

How often is the surface watered per month during winter?

[ ]

Is manure removed from the surface?

○ Yes

○ No

Is the arena salted?

○ Yes

○ No

Major maintenance/renovation:

How often to do think that top-layer renovation is needed?

○ Once a year or more often

- Every other to every fourth year
- Every fifth to every tenth year
- More seldom than every tenth year

What was the ingredients of the last renovation, or the next planned, of?

- Sand-mineral (from nature)
- Wood chip
- Fibre
- Rubber
- Saw dust
- Crushed material (stone dust)

OTHER

Typical expected useful lifetime of the arena surface. For example, how many years do you think the arena can be used before it has to be replaced?

- 1-3 years
- 4-6y ears
- 7-9 years
- 10-12 years
- 13-15 years
- Longer than 15 years

What yearly cost is reserved for the arena?

- 0-1000 SEK
- 1000-5000 SEK
- 5000-10'000 SEK
- 10'000-20'000 SEK
- 20'000-30'000 SEK
- 30'000-40'000 SEK
- 40'000-50'000 SEK
- 50'000 SEK or more

Are you planning to dispose of the worn-out material from your current arenas?

- Yes
- No

- ○ Don’t know
