# Supplementary material for: The Descriptions and Attitudes of Riders and Arena Owners to 656 Equestrian Sport Surfaces in Sweden
Source: Front Vet Sci. 2021 Dec 23;8:798910. doi: 10.3389/fvets.2021.798910 (PMC8732755; doi:10.3389/fvets.2021.798910)
Supplement: Supplementary file 4 [file Data_Sheet_4.pdf]

Supplementary Data 4:
Descriptive statistics of the span of desired property values for training and competition surface from a questionnaire on riding arenas in Sweden 2014.

Discipline	Property	Category	N	Mean	STD	10th Pctl	50th Pctl	90th Pctl
Dressage	Impact firmness	Competition: From	1417	2.5	0.5	2.0	2.5	3.0
		Competition: To	1404	3.3	0.5	3.0	3.2	4.0
		Training: From	1370	2.4	0.5	2.0	2.5	3.0
		Training: To	1364	3.4	0.6	3.0	3.2	4.0
	Cushioning	Competition: From	1201	3.1	0.6	2.5	3.0	4.0
		Competition: To	1194	4.0	0.6	3.0	4.0	5.0
		Training: From	1171	3.0	0.6	2.0	3.0	4.0
		Training: To	1163	4.1	0.6	3.2	4.0	5.0
	Responsiveness	Competition: From	1085	3.2	0.7	2.0	3.0	4.0
		Competition: To	1072	4.3	0.7	3.0	4.3	5.0
		Training: From	1061	3.0	0.7	2.0	3.0	4.0
		Training: To	1050	4.3	0.7	3.2	4.2	5.0
	Grip	Competition: From	1064	3.8	0.7	3.0	4.0	5.0
		Competition: To	1049	4.6	0.5	4.0	5.0	5.0
		Training: From	1045	3.7	0.7	3.0	3.9	4.7
		Training: To	1032	4.6	0.5	4.0	5.0	5.0
	Uniformity	Competition: From	1056	4.6	0.7	4.0	5.0	5.0
		Competition: To	1037	4.9	0.3	5.0	5.0	5.0
		Training: From	1039	4.3	0.9	3.0	4.6	5.0
		Training: To	1024	4.9	0.3	5.0	5.0	5.0
Show-jumping	Impact firmness	Competition: From	1449	2.6	0.6	2.0	2.7	3.0
		Competition: To	1439	3.6	0.5	3.0	3.5	4.0
		Training: From	1404	2.3	0.6	2.0	2.2	3.0
		Training: To	1398	3.5	0.6	3.0	3.5	4.0
	Cushioning	Competition: From	1192	3.1	0.6	2.0	3.0	4.0
		Competition: To	1178	4.1	0.6	3.1	4.0	5.0
		Training: From	1163	3.0	0.6	2.0	3.0	4.0
		Training: To	1149	4.1	0.6	3.4	4.0	5.0
	Responsiveness	Competition: From	1045	3.2	0.8	2.0	3.0	4.0
		Competition: To	1032	4.4	0.7	3.5	4.8	5.0
		Training: From	1027	3.0	0.8	2.0	3.0	4.0
		Training: To	1014	4.4	0.6	3.5	4.5	5.0
	Grip	Competition: From	1036	3.9	0.7	3.0	4.0	5.0
		Competition: To	1008	4.7	0.5	4.0	5.0	5.0
		Training: From	1011	3.6	0.8	3.0	3.8	4.6
		Training: To	992	4.7	0.5	4.0	5.0	5.0
	Uniformity	Competition: From	1026	4.3	0.9	3.0	4.8	5.0
		Competition: To	1004	4.9	0.4	5.0	5.0	5.0
		Training: From	1003	4.0	1.1	2.0	4.0	5.0
		Training: To	988	4.9	0.4	4.0	5.0	5.0
Eventing	Impact firmness	Competition: From	147	2.5	0.5	2.0	2.5	3.0
		Competition: To	144	3.5	0.5	3.0	3.5	4.0
		Training: From	144	2.2	0.6	1.0	2.0	3.0
		Training: To	145	3.7	0.7	3.0	3.5	5.0
	Cushioning	Competition: From	127	3.1	0.6	2.2	3.0	4.0
		Competition: To	127	4.0	0.5	3.2	4.0	5.0
		Training: From	127	2.8	0.7	2.0	3.0	3.7
		Training: To	127	4.2	0.5	3.5	4.0	5.0
	Responsiveness	Competition: From	117	3.0	0.6	2.0	3.0	3.8
		Competition: To	117	4.3	0.6	3.6	4.2	5.0
		Training: From	117	2.7	0.7	2.0	3.0	3.5

	Training: To	117	4.4	0.6	3.5	4.5	5.0
Grip	Competition: From	116	3.1	0.9	2.0	3.0	4.0
	Competition: To	116	4.5	0.6	4.0	4.5	5.0
	Training: From	116	2.9	0.9	2.0	3.0	4.0
	Training: To	116	4.5	0.5	4.0	4.5	5.0
Uniformity	Competition: From	116	3.8	1.1	2.0	4.0	5.0
	Competition: To	115	4.8	0.5	4.0	5.0	5.0
	Training: From	116	3.3	1.2	2.0	3.0	5.0
	Training: To	116	4.8	0.6	4.0	5.0	5.0
